# Nickel-Catalyzed Aminocarbonylation of Aryl Trifluoromethoxides

**DOI:** 10.1021/acs.orglett.6c00974

**Published:** 2026-03-26

**Authors:** Zhen-Wei Liu, Chang-Sheng Kuai, Xiao-Feng Wu

**Affiliations:** † Dalian National Laboratory for Clean Energy, Dalian Institute of Chemical Physics, Chinese Academy of Sciences, Dalian 116023, China; ‡ Leibniz-Institut für Katalyse e. V., 18059 Rostock, Germany

## Abstract

Aryl trifluoromethoxides (ArOCF_3_) are widely employed in pharmaceuticals and agrochemicals, which leads to their transformation also being attractive. Here we report a nickel-catalyzed aminocarbonylation of ArOCF_3_ with amines using inositol hexaformate (HFI) as the CO source, providing a potential approach for the chemical upcycling of these persistent compounds into value-added benzamide derivatives. The reaction exhibits broad compatibility with diverse aniline derivatives, accommodating various electronic and steric demands. This work not only expands the aryl source for amide synthesis from conventional electrophiles to the highly robust ArOCF_3_ motif but also offers a blueprint for phenol activation.

## Graphical abstract

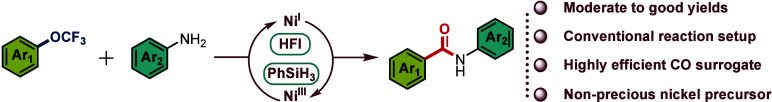

The aryl trifluoromethoxide (ArOCF_3_) motif is a privileged structure in modern pharmaceutical and agrochemical discovery, prized for the unique combination of high lipophilicity, strong electron-withdrawing character, and exceptional metabolic inertness offered by the trifluoromethoxy group (Scheme 1A).^1–5^ Yet this chemical “double-edged sword” carries an unintended environmental consequence: the very inertness that underpins its desirable traits now raises concerns over persistence (Scheme 1B).^6–9^ Addressing the challenges outlined above, the development of mild and selective methods for cleaving the C­(sp^2^)–OCF_3_ bond in ArOCF_3_ offers not only a promising strategy for remediating persistent pollutants but also an opportunity for the chemical upcycling of waste aromatic resources into value-added products.^10–13^ However, such transformations are inherently challenging. The bond dissociation energy (BDE) of the C­(sp^2^)–OCF_3_ bond is reported to be approximately 110 kcal/mol, ranking among the strongest C­(sp^2^)–heteroatom bonds known.^14^ This value substantially exceeds those of C­(sp^2^)–Br (82 kcal/mol), C­(sp^2^)–Cl (97 kcal/mol), and even the C­(sp^2^)–OCH_3_ bond in anisole (102 kcal/mol) (Scheme 1C). Consequently, the development of catalytic systems capable of overcoming these thermodynamics represents a critical step toward the resourceful utilization of ArOCF_3_-bearing waste materials.

**Scheme 1:**
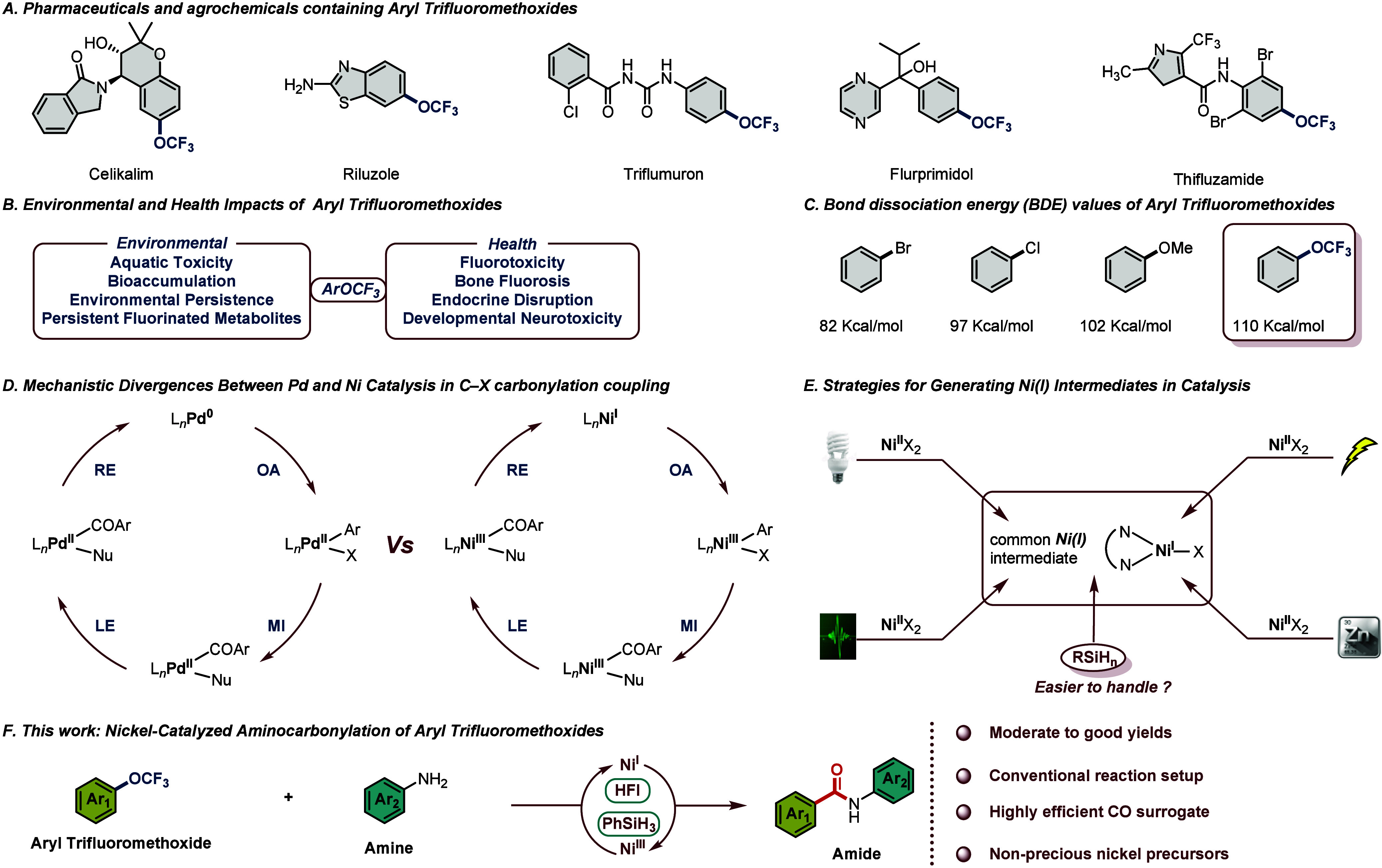
Chemistry of Aryl Trifluoromethoxides and Nickel Catalyst

Transition-metal catalysis has emerged as a powerful platform for carbonylative transformations of inert C–O bonds.^15–20^ Palladium catalysts, despite their widespread application in carbonylative cross-coupling reactions, are inherently limited by their Pd(0)/Pd­(II) two-electron redox cycles.^21–26^ This mechanistic constraint renders them ill-equipped for oxidative addition into the exceptionally strong C–OCF_3_ bond. Moreover, in carbonylative transformations, the coordination of carbon monoxide to the metal center attenuates electron density, further diminishing oxidative addition capacity, a challenge particularly acute for highly stable substrates such as ArOCF_3_.^27^ This limitation prompted us to explore alternative catalyst systems.

In contrast, nickel catalysts offer distinct advantages in this challenging carbonylative transformation. The electron-rich nature and smaller atomic radius of nickel species facilitate insertion into inert C–O bonds under carbonylative conditions.^28,29^ More distinctively, nickel catalysis is not confined to conventional Ni(0)/Ni­(II) pathways; it can also involve Ni­(I)/Ni­(III) manifolds via single-electron redox processes (Scheme 1D).^30–33^ This valence diversity may provide additional mechanistic channels and operational flexibility for activating strong bonds in the presence of CO, an advantage that is particularly pertinent, given the electron-withdrawing effect of CO coordination discussed above. Moreover, the substantially lower cost of nickel relative to palladium offers a distinct advantage in terms of sustainability, aligning with the principles of green chemistry and resource utilization.

In nickel-catalyzed C­(sp^2^)–heteroatom cross-couplings, Ni­(I) species are frequently invoked as key intermediates, spurring the development of efficient methods for their generation.^34^ Current approaches include photoredox catalysis,^35–38^ electrochemical reduction,^39–41^ pulse radiolysis,^42^ and the use of heterogeneous, preactivated zinc metal (Scheme 1E).^43,44^ However, these methods often suffer from limitations ranging from specialized equipment requirements to heterogeneous reaction conditions. Organosilanes have recently emerged as particularly attractive alternatives: they reduce Ni­(II) to catalytically active Ni­(I) species under mild, homogeneous conditions, offering operational simplicity while circumventing the heterogeneity inherent to metallic reductants.^45,46^

The benzamide scaffold is ubiquitous in pharmaceuticals, and aminocarbonylation offers an ideal choice.^47–50^ However, the use of aryl trifluoromethyl ethers as substrates remains unprecedented. Herein, we report the first nickel-catalyzed aminocarbonylation of ArOCF_3_ with amines, enabled by a silane reduction strategy (Scheme 1F). This work offers a potential approach for the chemical upcycling of environmentally persistent pollutants and expands the aryl source for benzamide synthesis to the ArOCF_3_ motif.

Due to the strong coordination tendency between nickel and carbon monoxide, nickel(0) species readily bind multiple CO ligands to form polycarbonyl complexes such as Ni­(CO)_4_.^29^ The electron density of these species is significantly depleted by the strong π-accepting ability of CO, which severely attenuates their capacity for oxidative addition to inert electrophiles and thereby impedes catalytic turnover. To address this issue, two complementary strategies have been developed. The first involves the design of sterically hindered multidentate ligands that preoccupy coordination sites on nickel, sterically preventing the binding of multiple CO ligands while preserving the electron-rich nature and reactivity of the nickel center.^51^ The second strategy employs CO surrogates that release CO slowly under reaction conditions, maintaining a low instantaneous CO concentration and thus circumventing overcoordination at the source.^52^

Building upon these insights, we selected the aminocarbonylation of 4-acetylphenyl trifluoromethyl ether with aniline as a model substrate to systematically optimize the reaction conditions (Table 1). Gratifyingly, the desired benzamide product **1** was obtained in 74% GC and 73% isolated yields under the following optimized conditions: NiCl_2_·6H_2_O as the catalyst, *p*-MeO-Phen as the ligand, phenylsilane as the reductant, HFI as the CO source, and DBU as the base, in NMP at 140 °C under a nitrogen atmosphere for 18 h (entry 1). The ligand structure proved critical for reaction efficiency. Replacing the 4,7-dimethoxy substituents on the phenanthroline backbone with methyl groups or hydrogen atoms led to a marked decrease in yield (entries 2, 3), suggesting that electron-rich ligands facilitate the oxidative addition of the nickel species into the C­(sp^2^)–OCF_3_ bond. When the rigid phenanthroline scaffold was replaced with the more flexible *p*-MeO-Bpy, a significant drop in yield was observed (entry 4), indicating that the ligand rigidity may be essential for maintaining catalytic activity. The nickel source also exerted a substantial influence. Substituting NiCl_2_·6H_2_O with less hygroscopic Ni­(acac)_2_, which is easier to handle under ambient conditions, resulted in a diminished yield of 60% (entry 5). Evaluation of alternative reductants revealed that inexpensive diphenylsilane afforded the product in 68% yield (entry 6), potentially due to increased steric hindrance impeding the reduction of Ni­(II). Notably, the greener homogeneous reductant B_2_pin_2_ delivered only trace amounts of the desired product (entry 7), underscoring the unique efficacy of silanes in this transformation. Base screening demonstrated that DBU was uniquely effective, which may also activate silane for nickel reduction; replacement with the organic weak base DIPEA or inorganic Na_2_CO_3_ resulted in negligible product formation (entries 8, 9). Further optimization revealed that solvent choice, reaction time, and temperature significantly impact catalytic performance (entries 10–13). Control experiments confirmed the essential roles of both the nickel catalyst and phenylsilane: no product was detected in their absence, lending support to the involvement of a Ni­(I) species as the catalytically active intermediate (entries 14, 15). It is worth mentioning that a noncarbonylation compound was the main byproduct detectable during the study.

**Table 1:**
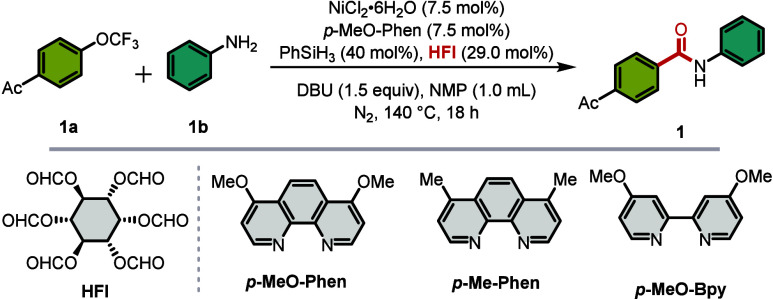
Screening of the Conditions^a^

Entry	Variations	Yield (%)
1	none	74 (73)^c^
2	*p*-Me-Phen	25
3	1,10-Phen	5
4	*p*-MeO-Phen	30
5	Ni(acac)_2_	60
6	Ph_2_SiH_2_	68
7	B_2_pin_2_	11
8	DIPEA	0
9	Na_2_CO_3_	8
10	MeCN	24
11	THF	0
12	12 h	37
13	130 °C	11
14	w/o NiCl_2_·6H_2_O	0
15	w/o PhSiH_3_	0

a Reactions were performed on a 0.2 mmol scale, aryl trifluoromethoxides (1.0 equiv), amine (3.0 equiv).

b Yields were determined by GC-MS analysis of the crude product using *n*-dodecane as the internal standard.

c Isolated yield.

With the optimized catalytic system in hand, we next explored the scope of this procedure (Scheme 2). In the examination of various aniline derivatives, the steric environment of the aniline coupling partner proved to have a pronounced effect on the reaction efficiency. As the steric bulk of *ortho*-substituents increased, a corresponding decrease in yield was observed (**1–3**, 73–49%). Notably, when the *ortho*-position bore a phenyl group or two methyl substituents, the desired products were obtained in less than 30% yield (**4**, **5**), underscoring the sensitivity of this transformation to steric congestion. In contrast, *para*-substituted anilines were well tolerated regardless of electronic nature: electron-donating groups (Me, Et, ^*t*^Bu, MeO) afforded the corresponding benzamides in good yields (**6**–**9**, 62–78%), and electron-withdrawing substituents such as fluoro and trifluoromethoxy groups also delivered similar yields (**10**, **11**, 47–79%). *meta*-Substituted anilines also participated smoothly, providing the desired products in good yields (**12–15**, 44–74%). Notably, the reaction exhibited excellent functional group tolerance: methoxy, trifluoromethoxy, and fluoro substituents were all compatible with the catalytic conditions (**9–11**, **15**, **16**, and **18**), offering handles for further synthetic elaboration. Disappointingly, anilines bearing polyaromatic motifs furnished only trace amounts of the desired products (**19**), highlighting the current limitations of the method. Polysubstituted anilines were generally well accommodated, delivering the corresponding benzamides in moderate yields (**5**, **17**, **18**, 27–47%).

**Scheme 2:**
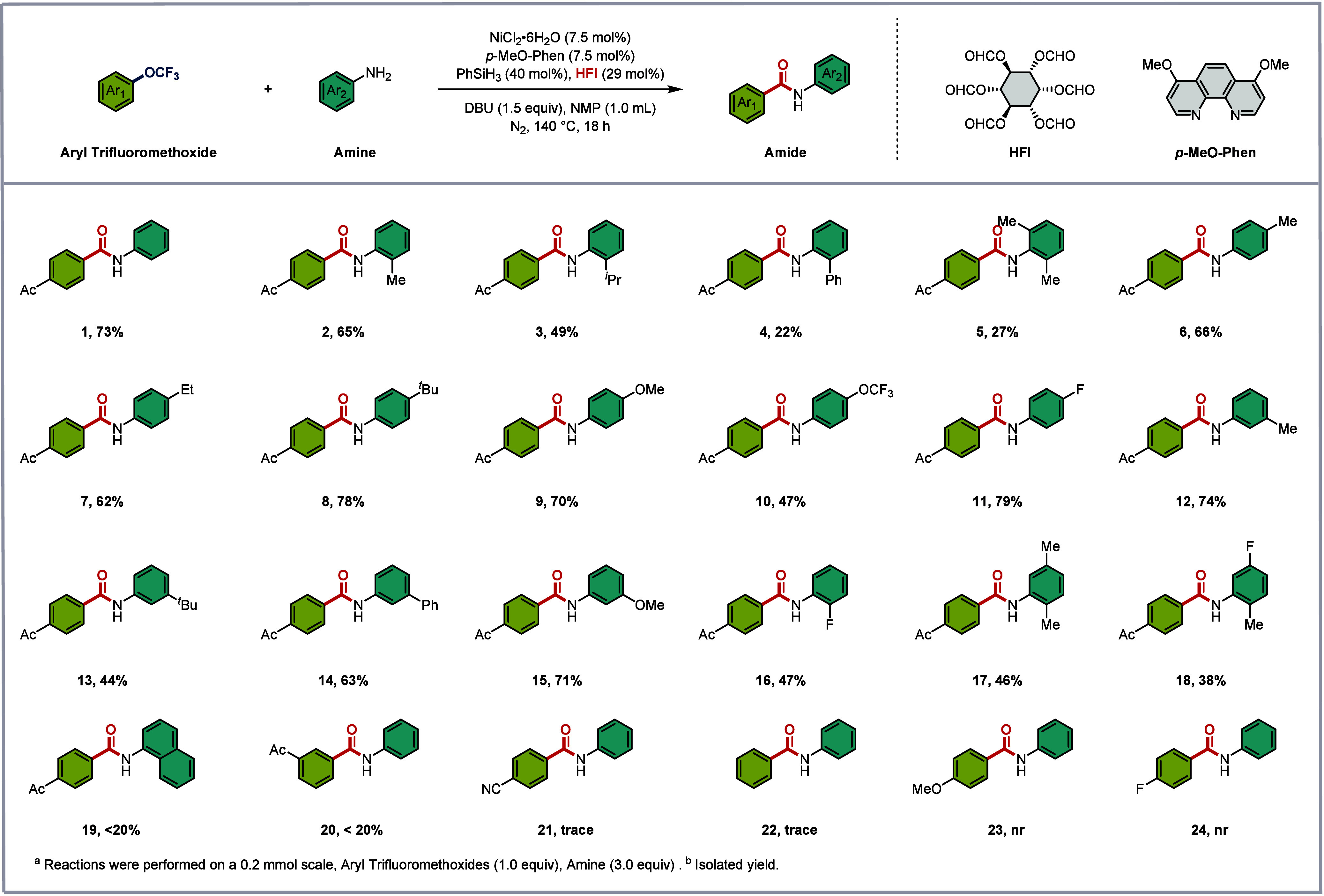
Scope of Substrates^*a*^

Encouraged by the broad compatibility with diverse anilines, we next turned our attention to the aryl trifluoromethyl ether component. Unfortunately, substrate variation on this coupling partner proved challenging: beyond the 4-acetyl-substituted model substrate, aryl trifluoromethyl ethers bearing electron-withdrawing groups or lacking substituents afforded only trace amounts of the desired products, while those bearing electron-donating groups or fluoro substituents failed to deliver any detectable product (**20**–**24**). Further details regarding unsuccessful substrates are provided in the Supporting Information (SI). These results indicate that the current catalytic system is highly sensitive to the electronic and structural features of the ArOCF_3_ coupling partner. Despite these limitations, this work represents a crucial proof-of-concept for nickel-catalyzed aminocarbonylation via C­(sp^2^)–OCF_3_ bond cleavage, offering a glimpse of the potential for upgrading persistent fluorinated pollutants into value-added benzamide derivatives.

To gain insight into the catalytic cycle of this transformation, a series of mechanistic experiments were conducted (Scheme 3). First, the origin of the carbonyl group in the amide product was probed. Under the standard reaction conditions, the desired benzamide was obtained in 73% yield. In contrast, when the reaction was performed in the absence of HFI, neither product was detectable. This result unequivocally establishes that the carbonyl moiety of the amide originates from HFI rather than from decomposition of the labile trifluoromethoxy anion (OCF_3_^–^) to carbonyl fluoride—a pathway that could potentially serve as an alternative CO source (Scheme 3A). Next, the oxidation state of the catalytically active nickel species was investigated. When Ni­(cod)_2_ was employed as the catalyst in the absence of silane, only trace amounts of the desired amide were detected. However, upon the addition of phenylsilane to the same Ni­(cod)_2_ precatalyst, the yield increased markedly to 41% (Scheme 3B).

**Scheme 3:**
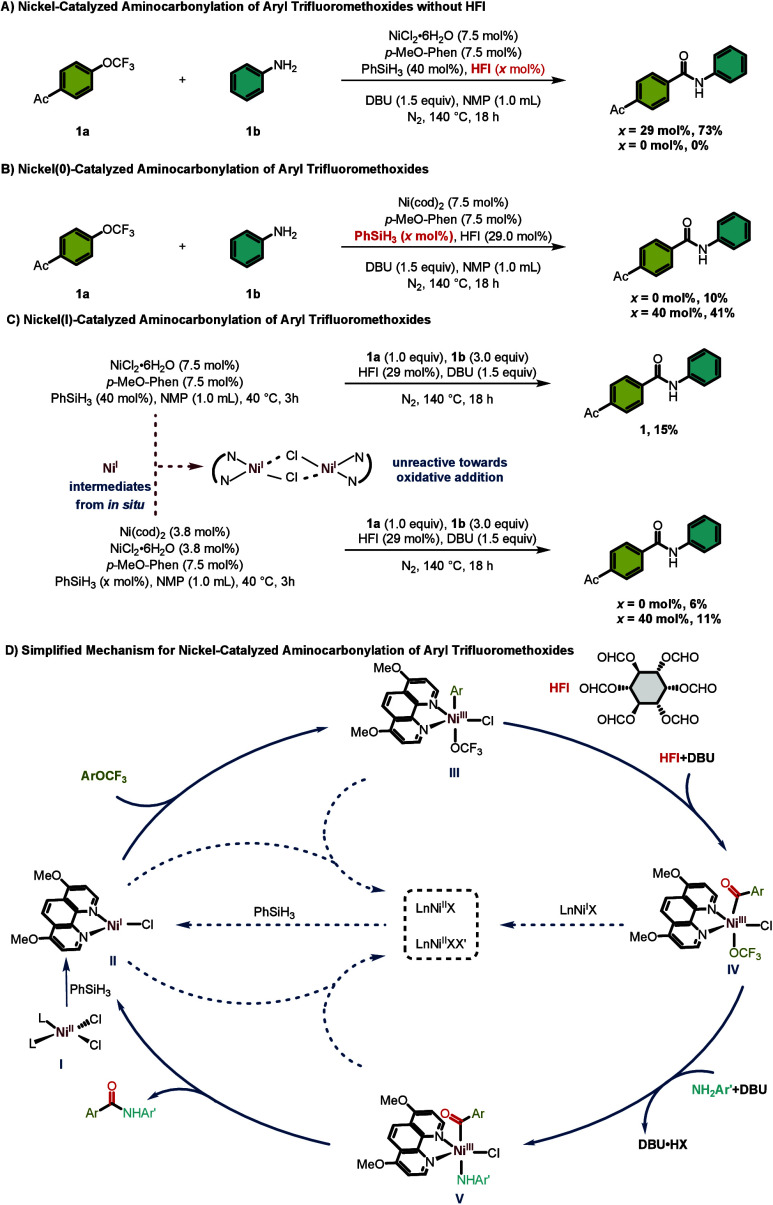
Control Experiments and Proposed Mechanism

This striking difference suggests that a Ni­(I) species, generated in situ via silane-mediated reduction of Ni­(II) or comproportionation between Ni(0) and Ni­(II), is likely the catalytically active species rather than Ni(0) itself. To further corroborate this hypothesis, a catalytic reaction employing an independently prepared Ni­(I) complex was conducted. Unexpectedly, only trace amounts of the desired product were observed under these conditions (Scheme 3C). This result may be rationalized by the tendency of Ni­(I) species to undergo rapid dimerization at elevated concentrations in the absence of electrophilic coupling partners, forming catalytically inactive dimers and thereby diminishing the yield. Collectively, these experiments support the involvement of a Ni­(I) intermediate while highlighting the critical role of silane in both generating and stabilizing the active nickel species throughout catalytic turnover.

Based on our results and literature,^53–55^ a plausible catalytic cycle is proposed (Scheme 3D). The cycle commences with reduction of Ni­(II) precursor **I** by phenylsilane to generate catalytically active Ni­(I) species **II**. This Ni­(I) intermediate then undergoes oxidative addition into the C­(sp^2^)–OCF_3_ bond of the aryl trifluoromethyl ether, affording Ni­(III) aryl species **III**. Concurrently, HFI decomposes under basic conditions at elevated temperature to release carbon monoxide, which coordinates to the nickel center of **III** and subsequently undergoes migratory insertion to yield the Ni­(III) acyl complex **IV**. In the presence of DBU, ligand exchange with the amine coupling partner furnishes the Ni­(III) intermediate **V**, which upon reductive elimination delivers the desired benzamide product and regenerates the Ni­(I) species **II** to re-enter the catalytic cycle. Notably, a competing disproportionation pathway may operate in parallel: the Ni­(I) species can undergo comproportionation with the Ni­(III) intermediate to generate the Ni­(II) complex. This off-cycle Ni­(II) species is subsequently reduced back to the active Ni­(I) catalyst by phenylsilane, thereby sustaining catalytic turnover. Throughout the cycle, phenylsilane plays a crucial role in initiating and maintaining the catalytic cycle through reduction of Ni­(II) to Ni­(I). The interplay between these pathways highlights the critical role of silane in enabling this challenging transformation.

In summary, we have developed the first nickel-catalyzed aminocarbonylation of aryl trifluoromethyl ethers. This transformation is enabled by a silane reduction strategy that generates catalytically active Ni­(I) species. The reaction exhibits broad compatibility with diverse aniline derivatives, accommodating various electronic and steric demands, although the scope of aryl trifluoromethyl ethers currently remains limited to activated substrates. This work not only offers a potential approach for the chemical upcycling of persistent fluorinated pollutants into value-added benzamide derivatives but also expands the aryl source for amide synthesis from conventional electrophiles to the highly robust ArOCF_3_ motif.

## Supplementary Material



## Data Availability

The data underlying this study are available in the published article and its Supporting Information.

## Notes

Experimental details, characterization data, and NMR spectra (PDF)
