# Supplementary material for: Nickel-Catalyzed Aminocarbonylation of Aryl Trifluoromethoxides
Source: Org Lett. 2026 Mar 26;28(14):4597–602. doi: 10.1021/acs.orglett.6c00974 (PMC13077685; doi:10.1021/acs.orglett.6c00974)
Supplement: Supplementary file 1 [file ol6c00974_si_001.pdf]

Supporting Information

Nickel-Catalyzed Aminocarbonylation of Aryl Trifluoromethoxides

Zhen-Wei Liu,[a,b] Chang-Sheng Kuai,[a] Xiao-Feng Wu*[a,b]

[a]. Dalian National Laboratory for Clean Energy, Dalian Institute of Chemical Physics, Chinese Academy of Sciences, Dalian 116023, China, E-mail: xwu2020@dicp.ac.cn

[b]. Leibniz-Institut für Katalyse e. V., 18059 Rostock, Germany

General Information ... 2

Safety Considerations ... 3

Optimization of conditions ... 4

Table S1. The results of the ligand ... 4
Table S2. The results of the nickel catalyst ... 5
Table S3. The results of the solvent ... 6
Table S4. The results of the HFI ... 7
Table S5. The results of the NiI_2•$6H_2O$ and *p*-MeO-Phen ... 8
Table S6. The results of the $PhSiH_3$ ... 9
Table S7. The results of the DBU ... 10
Table S8. The results of the temperature ... 11
Table S9. The results of the time ... 11

Experimental procedures ... 12

Unsuccessful examples ... 13

Analytical data for Products ... 14

References ... 23

NMR Spectra ... 24

General Information

Unless otherwise noted, all reactions were performed under positive pressure of nitrogen atmosphere in oven-dried flasks. All commercially available reagents were purchased from commercial vendors Sigma-Aldrich, Adamas, or Energy Chemical and used without further purification. Reactions were monitored by thin layer chromatography (TLC) (HaiYang, QingDao, China), visualized by UV (254 nm) and phosphomolybdic acid (PMA) staining. Flash column chromatography was performed on silica gel (200 ~ 300 mesh) purchased from Haiyang (Qingdao, China) and using petroleum ether (b.p. 60-90 °C) and ethyl acetate as the eluents.

Gas chromatography (**GC**) analyses were performed on an Agilent HP-7890A instrument with an FID detector and HP-5 capillary column (polydimethylsiloxane with 5% phenyl groups, 30 m, 0.32 mm i.d. 0.25 μm film thickness) using argon as carrier gas. Gas chromatography mass spectrometer (**GC-MS**) analyses were performed on a Shimadzu QP2020 NX instrument. **NMR** spectra were recorded on Bruker AVANCE III 400 MHz and Bruker AVANCE III 700 MHz. Chemical shifts (δ) were reported in ppm relative to residual solvent peak or tetramethylsilane as internal standard ($(CD_3)_2SO$: 2.50 ppm for ^1H NMR, 39.52 ppm for ^{13}C NMR). Multiplicity and qualifier abbreviations are as follows: s = singlet, d = doublet, t = triplet, q = quartets, dd = doublet of doublets, ddd = doublet of doublet of doublets, dddd = doublet of doublet of doublet of doublets, dt = doublet of triplets, dq = doublet of quartets, ddq = doublet of doublet of quartets, td = triplet of doublets, qd = quartet of doublets, m = multiplet. Highresolution mass spectral analysis (**HRMS**) data were determined on an Agilent 8890-7250 and Agilent Q-TOF 6540 spectrometer by means of ESI technique.

Safety Considerations

Carbon monoxide:

Carbon monoxide is highly toxic. The material should be handled by trained and experienced researchers, utilizing physical control (fume hoods) and safety measures (gas sensors). Experiments involving CO were conducted with a personal monitor worn on the researcher's lab coat at all times and an additional monitor placed near the regulator of the CO cylinder.

Nickel tetracarbonyl:

Extremely toxic and volatile (b.p. 43 ºC) $Ni(CO)_4$ could be potentially generated during nickel-catalyzed carbonylation reactions. Reactors which may contain $Ni(CO)_4$ should be handled by trained and experienced researchers, utilizing physical controls (fume hoods, chemically-resistant gloves) and safety measures (gas sensors). Any gas and solution that could potentially contain $Ni(CO)_4$ was quenched with a solution of iodine in acetone. After the desired reaction time, the reactors were allowed to cool to approximately 0 °C in an ice bath, after which 0.1 mL solutions of 20 wt% iodine in acetone were added (to quench any $Ni(CO)_4$). The mixture should then be stirred until it has warmed to room temperature. The generation of carbon monoxide (CO) has been observed to occur during this specific period.

Optimization of conditions

Table S1. The results of the ligand

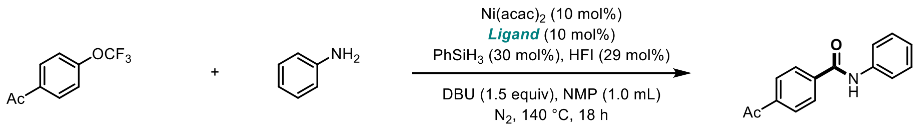

Aryl Trifluoromethoxide **Aniline** **1**

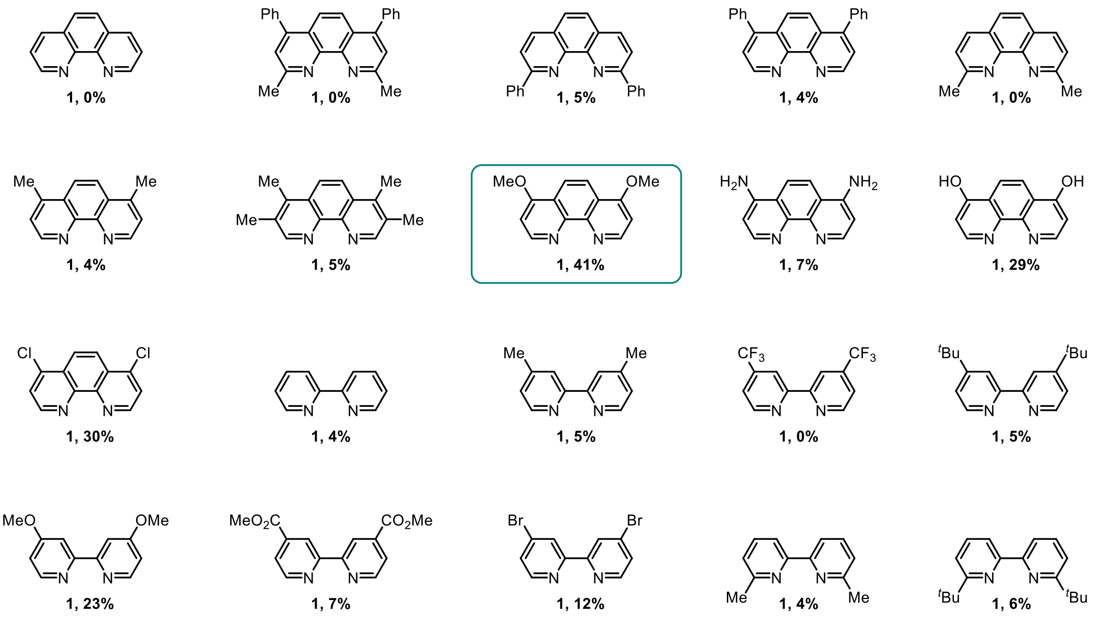

[a]Reaction conditions: Aryl Trifluoromethoxide (0.2 mmol, 1.0 equiv), aniline (4.0 equiv), $Ni(acac)_2$ (10 mol%), Ligand (10 mol%), $PhSiH_3$ (30 mol%), HFI (29 mol%), DBU (1.5 equiv), NMP (1.0 mL), 140 ^{o}C, 18 h. Yield was determined by GC-MS analysis of the crude product using *n*-dodecane as the internal standard.

Table S2. The results of the nickel catalyst

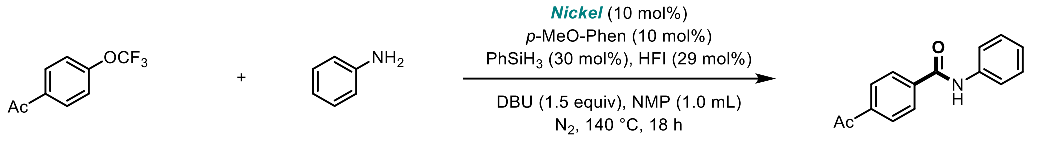

Entry	Nickel	Yield (%)
1	NiF_2	32
2	$NiCl_2$	38
3	$NiBr_2$	50
4	NiI_2	42
5	$NiF_2·4H_2O$	27
6	$NiCl_2·6H_2O$	53
7	$NiBr_2·3H_2O$	51
8	$NiI_2·6H_2O$	50
9	$Ni(BF_4)_2·6H_2O$	27
10	$Ni(ClO_4)_2·6H_2O$	38
11	$Ni(OAc)_2·6H_2O$	31
12	$Ni(NO_3)_2·6H_2O$	26
13	$NiCl_2·DME$	48
14	$NiBr_2·DME$	37
15	$Ni(acac)_2$	47
16	$Ni(TfO)_2$	45
17	$Ni(HFacac)_2$	19
18	$Ni(cod)_2$	32

[a]Reaction conditions: Aryl Trifluoromethoxide (0.2 mmol, 1.0 equiv), aniline (4.0 equiv), Nickel (10 mol%), *p*-MeO-Phen (10 mol%), $PhSiH_3$ (30 mol%), HFI (29 mol%), DBU (1.5 equiv), NMP (1.0 mL), 140 °C, 18 h. Yield was determined by GC-MS analysis of the crude product using *n*-dodecane as the internal standard.

Table S3. The results of the solvent

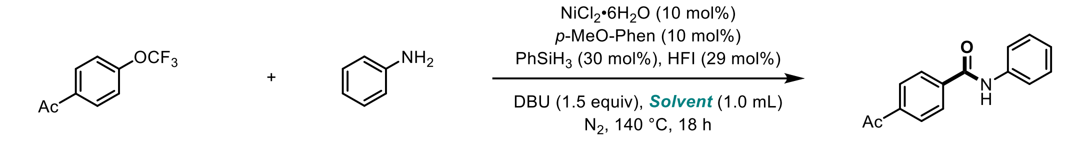

Entry	Solvent	Yield (%)
1	DMSO	0
2	DMAc	19
3	DMF	51
4	NMP	51
5	MeCN	18
6	THF	0
7	Dioxane	0
8	DME	0
9	CF_3Ph	0

[a]Reaction conditions: Aryl Trifluoromethoxide (0.2 mmol, 1.0 equiv), aniline (4.0 equiv), $NiCl_2•6H_2O$ (10 mol%), *p*-MeO-Phen (10 mol%), $PhSiH_3$ (30 mol%), HFI (29 mol%), DBU (1.5 equiv), Solvent (1.0 mL), 140 ^oC, 18 h. Yield was determined by GC-MS analysis of the crude product using *n*-dodecane as the internal standard. NMP has a higher boiling point than DNF, making it safer.

Table S4. The results of the HFI

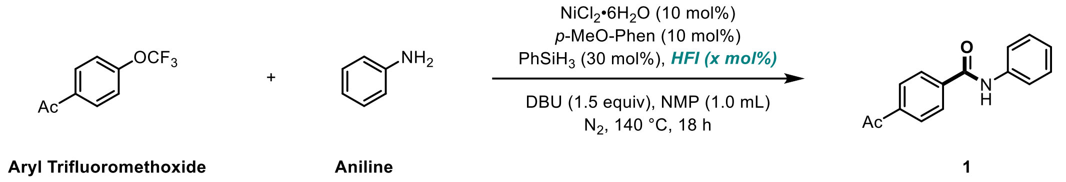

Entry	HFI (x mol%)	Yield (%)
1	17	32
2	21	44
3	25	49
4	29	55
5	33	49
6	38	49
7	42	34
8	46	26
9	50	21

[a]Reaction conditions: Aryl Trifluoromethoxide (0.2 mmol, 1.0 equiv), aniline (4.0 equiv), $NiCl_2$•$6H_2O$ (10 mol%), *p*-MeO-Phen (10 mol%), $PhSiH_3$ (30 mol%), HFI (x mol%), DBU (1.5 equiv), NMP (1.0 mL), 140 °C, 18 h. Yield was determined by GC-MS analysis of the crude product using *n*-dodecane as the internal standard.

Table S5. The results of the NiI_2•$6H_2O$ and *p*-MeO-Phen

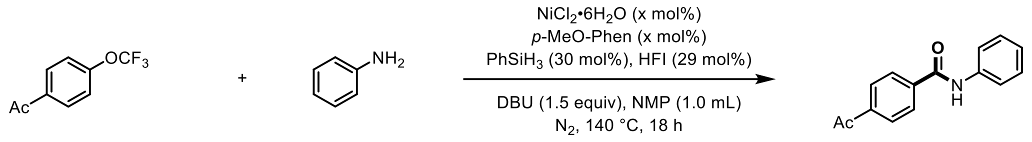

Entry	Catalyst (x mol%)	Yield (%)
1	2.5	12
2	5.0	19
3	7.5	66
4	10.0	66
5	12.5	65
6	15.0	58

[a]Reaction conditions: Aryl Trifluoromethoxide (0.2 mmol, 1.0 equiv), aniline (4.0 equiv), $NiCl_2$•$6H_2O$ (x mol%), *p*-MeO-Phen (x mol%), $PhSiH_3$ (30 mol%), HFI (29 mol%), DBU (1.5 equiv), NMP (1.0 mL), 140 °C, 18 h. Yield was determined by GC-MS analysis of the crude product using *n*-dodecane as the internal standard.

Table S6. The results of the $PhSiH_3$

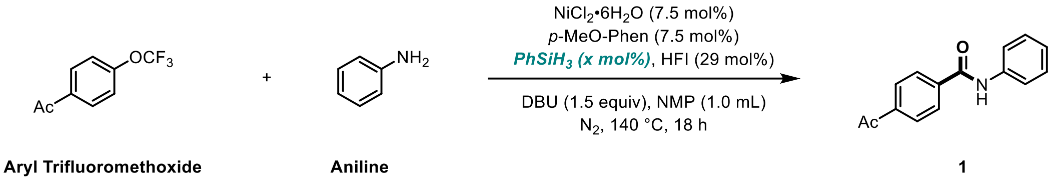

Entry	$PhSiH_3$ (x mol%)	Yield (%)
1	10	59
2	20	65
3	25	58
4	30	70
5	35	39
6	40	74
7	50	67
8	60	40
9	70	42
10	80	6
11	90	3
12	100	12

[a]Reaction conditions: Aryl Trifluoromethoxide (0.2 mmol, 1.0 equiv), aniline (4.0 equiv), $NiCl_2$•$6H_2O$ (7.5 mol%), *p*-MeO-Phen (7.5 mol%), $PhSiH_3$ (x mol%), HFI (29 mol%), DBU (1.5 equiv), NMP (1.0 mL), 140 °C, 18 h. Yield was determined by GC-MS analysis of the crude product using *n*-dodecane as the internal standard.

Table S7. The results of the DBU

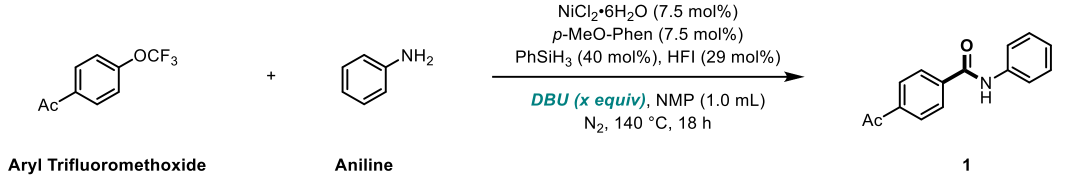

Entry	DBU (x equiv)	Yield (%)
1	1.0	53
2	1.5	72
3	2.0	63
4	2.5	61
5	3.0	62
6	3.5	63

[a]Reaction conditions: Aryl Trifluoromethoxide (0.2 mmol, 1.0 equiv), aniline (4.0 equiv), $NiCl_2$•$6H_2O$ (7.5 mol%), *p*-MeO-Phen (7.5 mol%), $PhSiH_3$ (40 mol%), HFI (29 mol%), DBU (x equiv), NMP (1.0 mL), 140 ^{o}C, 18 h. Yield was determined by GC-MS analysis of the crude product using *n*-dodecane as the internal standard.

Table S8. The results of the temperature

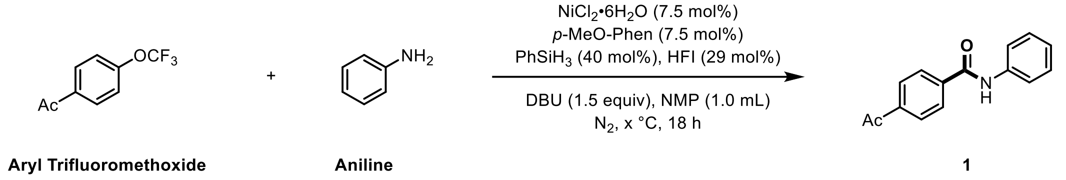

Entry	x °C	Yield (%)
1	120	0
2	130	11
3	140	74
4	150	62

[a]Reaction conditions: Aryl Trifluoromethoxide (0.2 mmol, 1.0 equiv), aniline (4.0 equiv), $NiCl_2•6H_2O$ (7.5 mol%), *p*-MeO-Phen (7.5 mol%), $PhSiH_3$ (40 mol%), HFI (29 mol%), DBU (1.5 equiv), NMP (1.0 mL), x °C, 18 h. Yield was determined by GC-MS analysis of the crude product using *n*-dodecane as the internal standard.

Table S9. The results of the time

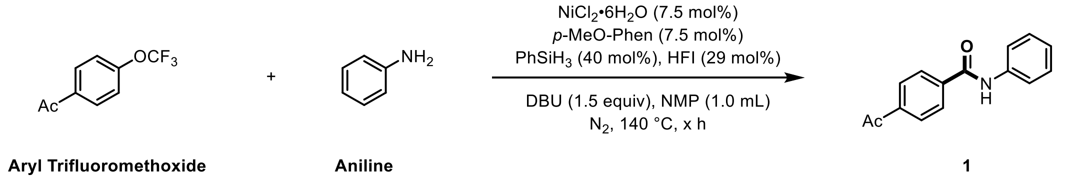

Entry	x h	Yield (%)
1	3	5
2	6	12
3	12	37
4	18	74
5	24	67

[a]Reaction conditions: Aryl Trifluoromethoxide (0.2 mmol, 1.0 equiv), aniline (4.0 equiv), $NiCl_2•6H_2O$ (7.5 mol%), *p*-MeO-Phen (7.5 mol%), $PhSiH_3$ (40 mol%), HFI (29 mol%), DBU (1.5 equiv), NMP (1.0 mL), 140 °C, x h. Yield was determined by GC-MS analysis of the crude product using *n*-dodecane as the internal standard.

Experimental procedures

General Procedure for Nickel-Catalyzed Aminocarbonylation of Aryl Trifluoromethoxides

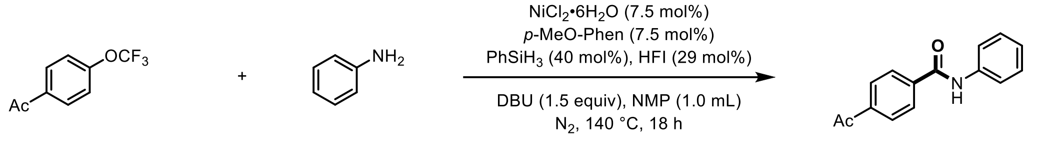

$NiCl_2$•$6H_2O$ (3.6 mg, 7.5 mol%), *p*-MeO-Phen (3.6 mg, 7.5 mol%) and HFI (20.2 mg, 29 mol%) were added to an oven-dried tube (15 mL) in Air. Then NMP (1.0 mL), aryl trifluoromethoxide (0.2 mmol, 1.0 equiv), aniline (3.0 equiv), DBU (45 μL, 1.5 equiv) and $PhSiH_3$ (10μL, 40 mol%) were added to the tube in glovebox. The tube was sealed and taken out of the glove box. The mixture was stirred at 140 °C (oil bath) with agitation at 500 rpm for 18 h in a shaker. After the reaction was completed, the reactors were allowed to cool to approximately 0 °C in an ice bath, after which 0.1 mL solutions of 20 wt% iodine in acetone were added (to quench any $Ni(CO)_4$). The mixture should then be stirred until it has warmed to room temperature. Ethyl acetate (5.0 mL) was added to it, and the reaction system was washed with 2.0 N aqueous hydrochloric acid (5.0 mL, 3 times) and then the aqueous phase was extracted with ethyl acetate (3 times). The collected organic phase was dried with anhydrous sodium sulfate, and concentrated by rotary evaporation. The crude product was purified by column chromatography on silica gel to afford the corresponding product amide.

1 mmol procedure: $NiCl_2$•$6H_2O$ (18 mg, 7.5 mol%), *p*-MeO-Phen (18 mg, 7.5 mol%) and HFI (101 mg, 29 mol%) were added to an oven-dried tube (15 mL) in Air. Then NMP (5.0 mL), 1-(4-(trifluoromethoxy)phenyl)ethan-1-one (1 mmol, 1.0 equiv), aniline (3.0 equiv), DBU (1.5 equiv) and $PhSiH_3$ (40 mol%) were added to the tube in glovebox. The tube was sealed and taken out of the glove box. The mixture was stirred at 140 °C (oil bath) with agitation at 500 rpm for 18 h in a shaker. After the reaction was completed, the reactors were allowed to cool to approximately 0 °C in an ice bath, after which 0.5 mL solutions of 20 wt% iodine in acetone were added (to quench any $Ni(CO)_4$). The mixture should then be stirred until it has warmed to room temperature. Ethyl acetate (5.0 mL) was added to it, and the reaction system was washed with 2.0 N aqueous hydrochloric acid (5.0 mL, 3 times) and then the aqueous phase was extracted with ethyl acetate (3 times). The collected organic phase was dried with anhydrous sodium sulfate, and concentrated by rotary evaporation. The crude product was purified by column chromatography on silica gel to afford the corresponding product amide **1** in 70% yield (167.3 mg).

Unsuccessful examples

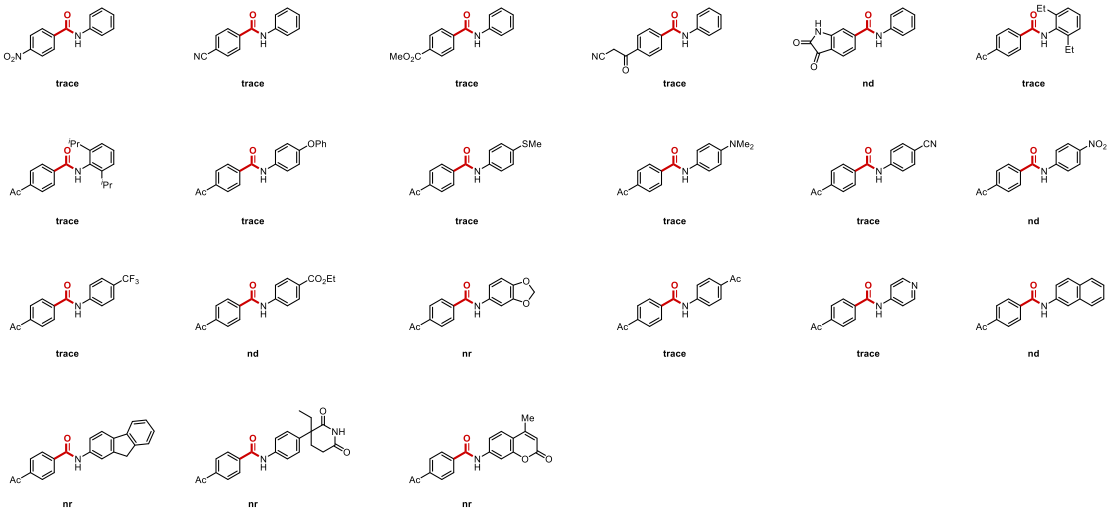

Analytical data for Products

4-Acetyl-*N*-phenylbenzamide (1)

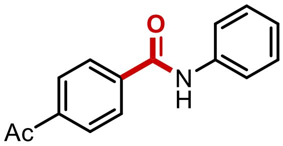

34.9 mg, white solid, yield: 73%.

R$_f$ = 0.3 (silica gel, petroleum ether : ethyl acetate : dichloromethane = 3:1:0.3)

^{1}H NMR (400 MHz, DMSO-*d$_6$*) δ 10.41 (s, 1H), 8.08 (d, *J* = 1.3 Hz, 4H), 7.87 – 7.70 (m, 2H), 7.37 (dd, *J* = 8.5, 7.4 Hz, 2H), 7.13 (td, *J* = 7.4, 1.2 Hz, 1H), 2.65 (s, 3H).

^{13}C NMR (101 MHz, DMSO-*d$_6$*) δ 197.7, 164.8, 138.9, 138.8, 138.8, 128.7, 128.2, 128.0, 124.0, 120.5, 27.0.[1]

Melting point: 135 – 138 °C.

4-Acetyl-*N*-(*o*-tolyl)benzamide (2)

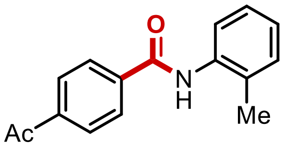

33.0 mg, white solid, yield: 65%.

R$_f$ = 0.4 (silica gel, petroleum ether : ethyl acetate : dichloromethane = 2:1:0.2)

^{1}H NMR (400 MHz, DMSO-*d$_6$*) δ 10.06 (s, 1H), 8.09 (s, 4H), 7.45 – 7.11 (m, 4H), 2.64 (s, 3H), 2.24 (s, 3H).

^{13}C NMR (101 MHz, DMSO-*d$_6$*) δ 197.7, 164.6, 138.9, 138.4, 136.1, 133.8, 130.4, 128.2, 128.0, 126.6, 126.2, 126.1, 27.0, 17.9.

HRMS (ESI-TOF) m/z: $[M+H]^+$ calcd for $C_{16}H_{16}NO_2^+$ 254.1176; found: 254.1170.

Melting point: 114 – 116 °C.

4-Acetyl-*N*-(2-isopropylphenyl)benzamide (3)

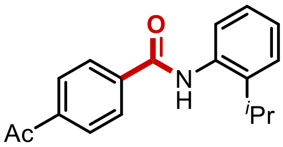

27.4 mg, white solid, yield: 49%.

R$_f$ = 0.3 (silica gel, petroleum ether : ethyl acetate : dichloromethane = 3:1:0.3)

^{1}H NMR (400 MHz, DMSO-d_6) δ 10.10 (s, 1H), 8.10 (s, 4H), 7.47 – 7.14 (m, 4H), 3.18 (hept, J = 6.8 Hz, 1H), 2.65 (s, 3H), 1.16 (d, J = 6.8 Hz, 6H).

^{13}C NMR (101 MHz, DMSO-d_6) δ 197.7, 165.2, 144.8, 138.9, 138.3, 134.7, 128.3, 128.1, 127.9, 127.1, 125.9, 125.7, 27.6, 27.0, 23.1.

HRMS (ESI-TOF) m/z: $[M+H]^+$ calcd for $C_{18}H_{20}NO_2^+$ 282.1489; found: 282.1480.

Melting point: 223 – 225 °C.

***N*-([1,1'-Biphenyl]-2-yl)-4-acetylbenzamide (4)**

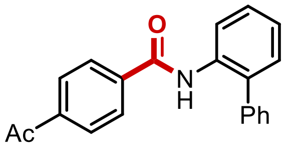

14.0 mg, white solid, yield: 22%.

R_f = 0.3 (silica gel, petroleum ether : ethyl acetate : dichloromethane = 5:1:0.5)

^{1}H NMR (400 MHz, DMSO-d_6) δ 10.04 (s, 1H), 8.01 (d, J = 8.4 Hz, 2H), 7.88 (d, J = 8.3 Hz, 2H), 7.57 – 7.18 (m, 9H), 2.61 (s, 3H).

^{13}C NMR (101 MHz, DMSO-d_6) δ 197.7, 165.0, 139.1, 138.8, 138.4, 138.3, 134.6, 130.3, 128.6, 128.4, 128.2, 128.1, 127.9, 127.7, 127.2, 127.0, 27.0.

HRMS (ESI-TOF) m/z: $[M+H]^+$ calcd for $C_{21}H_{18}NO_2^+$ 316.1332; found: 316.1323.

Melting point: 125 – 127 °C.

4-Acetyl-*N*-(2,6-dimethylphenyl)benzamide (5)

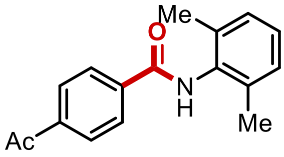

14.6 mg, white solid, yield: 27%.

R_f = 0.3 (silica gel, petroleum ether : ethyl acetate : dichloromethane = 3:1:0.3)

^{1}H NMR (400 MHz, DMSO-d_6) δ 9.95 (s, 1H), 8.10 (d, J = 2.6 Hz, 4H), 7.14 (s, 3H), 2.65 (s, 3H), 2.19 (s, 6H).

^{13}C NMR (101 MHz, DMSO-d_6) δ 197.7, 164.3, 138.9, 138.2, 135.6, 135.0, 128.3, 127.8, 127.8, 126.8, 27.0, 18.0.

HRMS (ESI-TOF) m/z: $[M+H]^+$ calcd for $C_{17}H_{18}NO_2^+$ 268.1332; found: 268.1320.

Melting point: 150 – 152 °C.

4-Acetyl-*N*-(*p*-tolyl)benzamide (6)

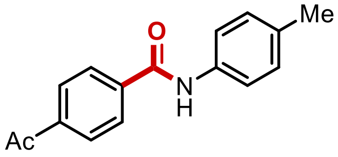

33.6 mg, white solid, yield: 66%.

R_f = 0.3 (silica gel, petroleum ether : ethyl acetate : dichloromethane = 3:1:0.3)

^{1}H NMR (400 MHz, DMSO-d_6) δ 10.33 (s, 1H), 8.07 (d, *J* = 1.6 Hz, 4H), 7.67 (d, *J* = 8.4 Hz, 2H), 7.17 (d, *J* = 8.4 Hz, 2H), 2.64 (s, 3H), 2.28 (s, 3H).

^{13}C NMR (101 MHz, DMSO-d_6) δ 197.7, 164.5, 138.8, 138.8, 136.4, 132.9, 129.0, 128.2, 127.9, 120.5, 27.0, 20.5.

HRMS (ESI-TOF) m/z: $[M+H]^+$ calcd for $C_{16}H_{16}NO_2^+$ 254.1176; found: 254.1174.

Melting point: 208 – 210 °C.

4-Acetyl-*N*-(4-ethylphenyl)benzamide (7)

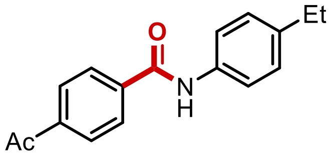

33.1 mg, white solid, yield: 62%.

R_f = 0.3 (silica gel, petroleum ether : ethyl acetate : dichloromethane = 3:1:0.3)

^{1}H NMR (400 MHz, DMSO-d_6) δ 10.34 (s, 1H), 8.08 (s, 4H), 7.69 (d, *J* = 8.6 Hz, 2H), 7.20 (d, *J* = 8.6 Hz, 2H), 2.64 (s, 3H), 2.58 (q, *J* = 7.6 Hz, 2H), 1.18 (t, *J* = 7.6 Hz, 3H).

^{13}C NMR (101 MHz, DMSO-d_6) δ 197.7, 164.5, 139.4, 138.8, 138.8, 136.6, 128.2, 128.0, 127.8, 120.5, 27.7, 27.0, 15.7.

HRMS (ESI-TOF) m/z: $[M+H]^+$ calcd for $C_{17}H_{18}NO_2^+$ 268.1332; found: 268.1329.

Melting point: 176 – 178 °C.

4-Acetyl-*N*-(4-(*tert*-butyl)phenyl)benzamide (8)

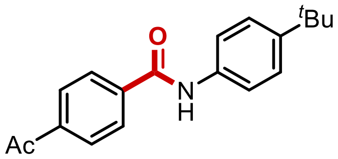

45.9 mg, white solid, yield: 78%.

R_f = 0.3 (silica gel, dichloromethane : ethyl acetate = 35:1)

^{1}H NMR (400 MHz, DMSO-d_6) δ 10.35 (s, 1H), 8.08 (s, 4H), 7.70 (d, J = 8.7 Hz, 2H), 7.38 (d, J = 8.7 Hz, 2H), 2.64 (s, 3H), 1.28 (s, 9H).

^{13}C NMR (101 MHz, DMSO-d_6) δ 197.7, 164.5, 146.3, 138.8, 138.8, 136.3, 128.2, 128.0, 125.2, 120.2, 34.1, 31.2, 27.0.[2]

Melting point: 171 – 173 °C.

4-Acetyl-*N*-(4-methoxyphenyl)benzamide (9)

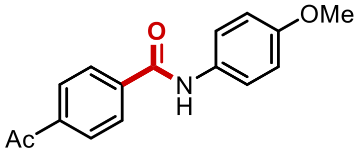

37.7 mg, white solid, yield: 70%.

R_f = 0.3 (silica gel, dichloromethane : ethyl acetate = 8:1)

^{1}H NMR (400 MHz, DMSO-d_6) δ 10.29 (s, 1H), 8.07 (s, 4H), 7.69 (d, J = 9.0 Hz, 2H), 6.94 (d, J = 9.0 Hz, 2H), 3.75 (s, 3H), 2.64 (s, 3H).

^{13}C NMR (101 MHz, DMSO-d_6) δ 197.7, 164.3, 155.7, 138.8, 138.7, 132.0, 128.2, 127.9, 122.1, 113.8, 55.2, 27.0.

HRMS (ESI-TOF) m/z: $[M+H]^+$ calcd for $C_{16}H_{16}NO_3^+$ 270.1125; found: 270.1121.

Melting point: 213 – 215 °C.

4-Acetyl-*N*-(4-(trifluoromethoxy)phenyl)benzamide (10)

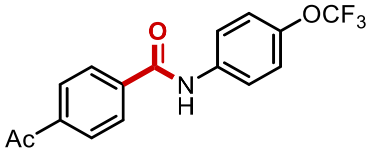

30.1 mg, white solid, yield: 47%.

R_f = 0.3 (silica gel, petroleum ether : ethyl acetate : dichloromethane = 3:1:0.3)

^{1}H NMR (400 MHz, DMSO-d_6) δ 10.59 (s, 1H), 8.16 – 8.02 (m, 4H), 7.91 (d, J = 9.2 Hz, 2H), 7.38 (d, J = 8.8 Hz, 2H), 2.65 (s, 3H).

^{13}C NMR (101 MHz, DMSO-d_6) δ 197.7, 164.9, 144.1, 144.0, 139.0, 138.4, 138.1, 128.2, 128.1, 121.8, 121.5, 120.2 (q, J_{C-F} = 225.7 Hz), 27.0.

^{19}F NMR (376 MHz, DMSO-d_6) δ -57.02.

HRMS (ESI-TOF) m/z: $[M+H]^+$ calcd for $C_{16}H_{13}F_3NO_3^+$ 324.0842; found: 324.0838.

Melting point: 201 – 203 °C.

4-Acetyl-*N*-(4-fluorophenyl)benzamide (11)

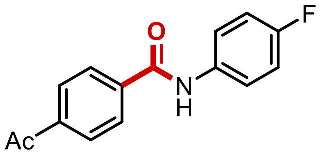

40.7 mg, white solid, yield: 79%.

R_f = 0.3 (silica gel, petroleum ether : ethyl acetate : dichloromethane = 2:1:0.2)

^{1}H NMR (400 MHz, DMSO-*d_6*) δ 10.47 (s, 1H), 8.19 – 7.98 (m, 4H), 7.89 – 7.72 (m, 2H), 7.21 (t, J = 8.9 Hz, 2H), 2.64 (s, 3H).

^{13}C NMR (101 MHz, DMSO-*d_6*) δ 197.7, 164.7, 158.4 (d, $J_{C\text{-}F}$ = 240.6 Hz), 138.9, 138.6, 135.3 (d, $J_{C\text{-}F}$ = 2.6 Hz), 128.2, 128.0, 122.3 (d, $J_{C\text{-}F}$ = 7.7 Hz), 115.3 (d, $J_{C\text{-}F}$ = 22.0 Hz), 27.0.

^{19}F NMR (376 MHz, DMSO-*d_6*) δ -118.51.

HRMS (ESI-TOF) m/z: $[M+H]^+$ calcd for $C_{15}H_{13}FNO_2^+$ 258.0925; found: 258.0919.

Melting point: 185 – 187 °C.

4-Acetyl-*N*-(m-tolyl)benzamide (12)

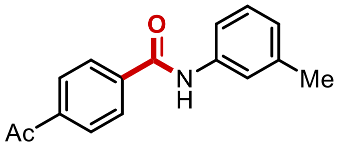

37.4 mg, white solid, yield: 74%.

R_f = 0.4 (silica gel, petroleum ether : ethyl acetate : dichloromethane = 3:1:0.3)

^{1}H NMR (400 MHz, DMSO-*d_6*) δ 10.33 (s, 1H), 8.08 (s, 4H), 7.69 – 7.50 (m, 2H), 7.24 (t, J = 7.8 Hz, 1H), 6.94 (d, J = 7.6 Hz, 1H), 2.64 (s, 3H), 2.32 (s, 3H).

^{13}C NMR (101 MHz, DMSO-*d_6*) δ 197.7, 164.7, 138.8, 138.8, 137.8, 128.5, 128.2, 128.0, 124.6, 121.0, 117.6, 27.0, 21.2.

HRMS (ESI-TOF) m/z: $[M+H]^+$ calcd for $C_{16}H_{16}NO_2^+$ 254.1176; found: 254.1173.

Melting point: 122 – 124 °C.

4-Acetyl-*N*-(3-(*tert*-butyl)phenyl)benzamide (13)

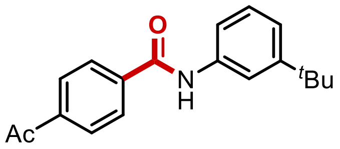

25.8 mg, colorless oil, yield: 44%.

R_f = 0.3 (silica gel, petroleum ether : ethyl acetate : dichloromethane = 4:1:0.4)

^{1}H NMR (400 MHz, DMSO-d_6) δ 10.34 (s, 1H), 8.08 (s, 4H), 7.80 (d, J = 1.8 Hz, 1H), 7.69 (dd, J = 7.9, 2.2 Hz, 1H), 7.29 (t, J = 7.9 Hz, 1H), 7.20 – 7.10 (m, 1H), 2.65 (s, 3H), 1.29 (s, 9H).

^{13}C NMR (101 MHz, DMSO-d_6) δ 197.7, 164.6, 151.2, 138.8, 138.8, 138.7, 128.3, 128.1, 128.0, 120.9, 117.6, 117.5, 34.5, 31.1, 27.0.

HRMS (ESI-TOF) m/z: $[M+H]^+$ calcd for $C_{19}H_{22}NO_2^+$ 296.1645; found: 296.1646.

Melting point: 112 – 114 °C.

***N*-([1,1'-Biphenyl]-3-yl)-4-acetylbenzamide (14)**

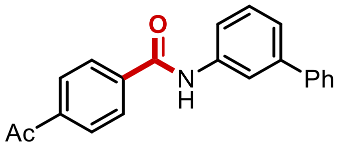

39.9 mg, white solid, yield: 63%.

R_f = 0.3 (silica gel, petroleum ether : ethyl acetate : dichloromethane = 3:1:0.3)

^{1}H NMR (400 MHz, DMSO-d_6) δ 10.51 (s, 1H), 8.12 (d, J = 7.0 Hz, 5H), 7.83 (dd, J = 7.8, 1.3 Hz, 1H), 7.69 – 7.63 (m, 2H), 7.53 – 7.34 (m, 5H), 2.65 (s, 3H).

^{13}C NMR (101 MHz, DMSO-d_6) δ 197.7, 164.8, 140.7, 140.1, 139.5, 138.9, 138.7, 129.3, 129.0, 128.2, 128.0, 127.6, 126.6, 122.3, 119.4, 118.8, 27.0.

HRMS (ESI-TOF) m/z: $[M+H]^+$ calcd for $C_{21}H_{18}NO_2^+$ 316.1332; found: 316.1332.

Melting point: 275 – 277 °C.

4-Acetyl-*N*-(3-methoxyphenyl)benzamide (15)

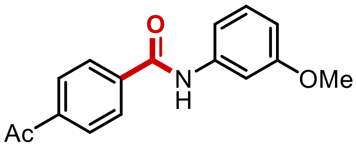

38.4 mg, white solid, yield: 71%.

R_f = 0.3 (silica gel, petroleum ether : ethyl acetate : dichloromethane = 3:1:0.3)

^{1}H NMR (400 MHz, DMSO-*d_6*) δ 10.38 (s, 1H), 8.17 – 7.98 (m, 4H), 7.48 (s, 1H), 7.39 (dd, J = 8.1, 2.0 Hz, 1H), 7.27 (t, J = 8.1 Hz, 1H), 6.71 (dd, J = 8.2, 2.7 Hz, 1H), 3.76 (s, 3H), 2.64 (s, 3H).

^{13}C NMR (101 MHz, DMSO-*d_6*) δ 197.7, 164.8, 159.4, 140.1, 138.8, 138.7, 129.4, 128.2, 128.0, 112.6, 109.4, 106.1, 55.0, 27.0.

HRMS (ESI-TOF) m/z: $[M+H]^+$ calcd for $C_{16}H_{16}NO_3^+$ 270.1125; found: 270.1121.

Melting point: 134 – 136 °C.

4-Acetyl-*N*-(2-fluorophenyl)benzamide (16)

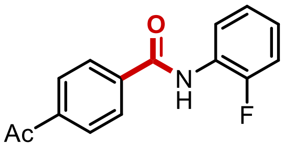

24.2 mg, white solid, yield: 47%.

R_f = 0.3 (silica gel, petroleum ether : ethyl acetate : dichloromethane = 4:1:0.4)

^{1}H NMR (400 MHz, DMSO-*d_6*) δ 10.31 (s, 1H), 8.10 (s, 4H), 7.62 (td, J = 7.9, 1.6 Hz, 1H), 7.40 – 7.17 (m, 3H), 2.65 (s, 3H).

^{13}C NMR (101 MHz, DMSO-*d_6*) δ 197.7, 164.7, 155.8 (d, $J_{C\text{-}F}$ = 247.2 Hz), 128.2, 128.1, 127.2 (d, $J_{C\text{-}F}$ = 2.2 Hz), 125.5 (d, $J_{C\text{-}F}$ = 12.5 Hz), 124.4 (d, $J_{C\text{-}F}$ = 3.7 Hz), 116.0 (d, $J_{C\text{-}F}$ = 19.8 Hz), 27.0.

^{19}F NMR (376 MHz, DMSO-*d_6*) δ -120.96.[1]

Melting point: 132– 134 °C.

4-Acetyl-*N*-(2,5-dimethylphenyl)benzamide (17)

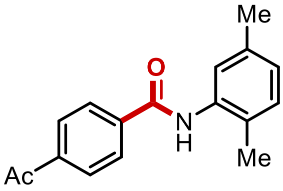

24.4 mg, white solid, yield: 46%.

R_f = 0.3 (silica gel, petroleum ether : ethyl acetate : dichloromethane = 4:1:0.4)

^{1}H NMR (400 MHz, DMSO-d_6) δ 10.00 (s, 1H), 8.09 (s, 4H), 7.16 (d, J = 7.8 Hz, 2H), 7.00 (d, J = 7.6 Hz, 1H), 2.64 (s, 3H), 2.29 (s, 3H), 2.19 (s, 3H).

^{13}C NMR (101 MHz, DMSO-d_6) δ 197.7, 164.5, 138.8, 138.4, 135.9, 135.1, 130.6, 130.2, 128.2, 127.9, 127.1, 126.9, 27.0, 20.5, 17.4.

HRMS (ESI-TOF) m/z: $[M+H]^+$ calcd for $C_{17}H_{17}NO_2^+$ 268.1332; found: 268.1330.

Melting point: 178 – 180 °C.

4-Acetyl-*N*-(5-fluoro-2-methylphenyl)benzamide (18)

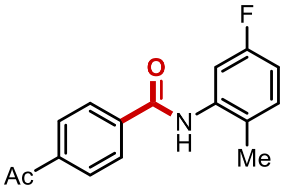

20.8 mg, white solid, yield: 38%.

R_f = 0.3 (silica gel, petroleum ether : ethyl acetate : dichloromethane = 3:1:0.3)

^{1}H NMR (400 MHz, DMSO-d_6) δ 10.08 (s, 1H), 8.09 (s, 4H), 7.42 – 7.18 (m, 2H), 7.03 (td, J = 8.5, 3.0 Hz, 1H), 2.65 (s, 3H), 2.23 (s, 3H).

^{13}C NMR (101 MHz, DMSO-d_6) δ 197.7, 164.7, 160.1 (d, J_{C-F} = 241.0 Hz), 139.0, 138.1, 137.4 (d, J_{C-F} = 10.3 Hz), 131.5 (d, J_{C-F} = 8.8 Hz), 129.2 (d, J_{C-F} = 2.9 Hz), 128.2, 128.1, 129.2 (d, J_{C-F} = 25.1 Hz), 112.6 (d, J_{C-F} = 20.5 Hz), 27.0, 17.2.

^{19}F NMR (376 MHz, DMSO-d_6) δ -116.95.

HRMS (ESI-TOF) m/z: $[M+H]^+$ calcd for $C_{16}H_{15}FNO_2^+$ 272.1081; found: 272.1077.

Melting point: 142– 144 °C.

4-Acetyl-*N*-(naphthalen-1-yl)benzamide (19)

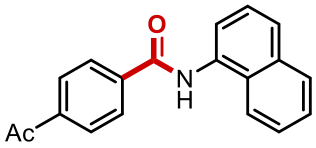

7.8 mg, white solid, yield: < 20%.

R_f = 0.3 (silica gel, petroleum ether : ethyl acetate : dichloromethane = 3:1:0.3)

^{1}H NMR (400 MHz, DMSO-*d_6*) δ 10.6 (s, 1H), 8.2 (d, *J* = 8.3 Hz, 2H), 8.1 (d, *J* = 8.3 Hz, 2H), 8.0 – 8.0 (m, 2H), 7.9 (d, *J* = 7.9 Hz, 1H), 7.6 (d, *J* = 7.0 Hz, 1H), 7.6 – 7.5 (m, 3H), 2.7 (s, 3H).

^{13}C NMR (101 MHz, DMSO-*d_6*) δ 197.8, 165.5, 139.0, 138.3, 133.8, 133.5, 129.1, 128.3, 128.2, 128.1, 126.5, 126.1, 126.1, 125.6, 123.9, 123.3, 27.0.

HRMS (ESI-TOF) m/z: $[M+H]^+$ calcd for $C_{19}H_{16}NO_2^+$ 290.1176; found: 290.1174.

Melting point: 195– 196 °C.

3-Acetyl-*N*-phenylbenzamide (20)

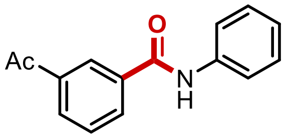

9.1 mg, white solid, yield: < 20%.

R_f = 0.3 (silica gel, petroleum ether : ethyl acetate : dichloromethane = 3:1:0.3)

^{1}H NMR (400 MHz, DMSO-*d_6*) δ 10.43 (s, 1H), 8.49 (s, 1H), 8.18 (ddt, *J* = 15.5, 7.7, 1.4 Hz, 2H), 7.78 (d, *J* = 8.7 Hz, 2H), 7.70 (t, *J* = 7.8 Hz, 1H), 7.37 (t, *J* = 7.9 Hz, 2H), 7.13 (td, *J* = 7.4, 1.1 Hz, 1H), 2.67 (s, 3H).

^{13}C NMR (101 MHz, DMSO-*d_6*) δ 197.6, 164.8, 139.0, 136.9, 135.4, 132.2, 131.1, 129.0, 128.7, 127.3, 123.9, 120.5, 26.9.

HRMS (ESI-TOF) m/z: $[M+H]^+$ calcd for $C_{15}H_{14}NO_2^+$ 240.1019; found: 240.1014.

Melting point: 132– 134 °C.

References

[1] H. Liu, L. Chen, F. Zhou, Y.-X. Zhang, J. Xu, M. Xu, S.-P. Bai, *Biorg. Med. Chem.* **2019**, *27*, 3089-3096.

[2] Z.-W. Liu, Y. Wang, R.-H. A, X.-F. Wu, *Chem. Sci.* **2025**, *16*, 23315-23320.

NMR Spectra

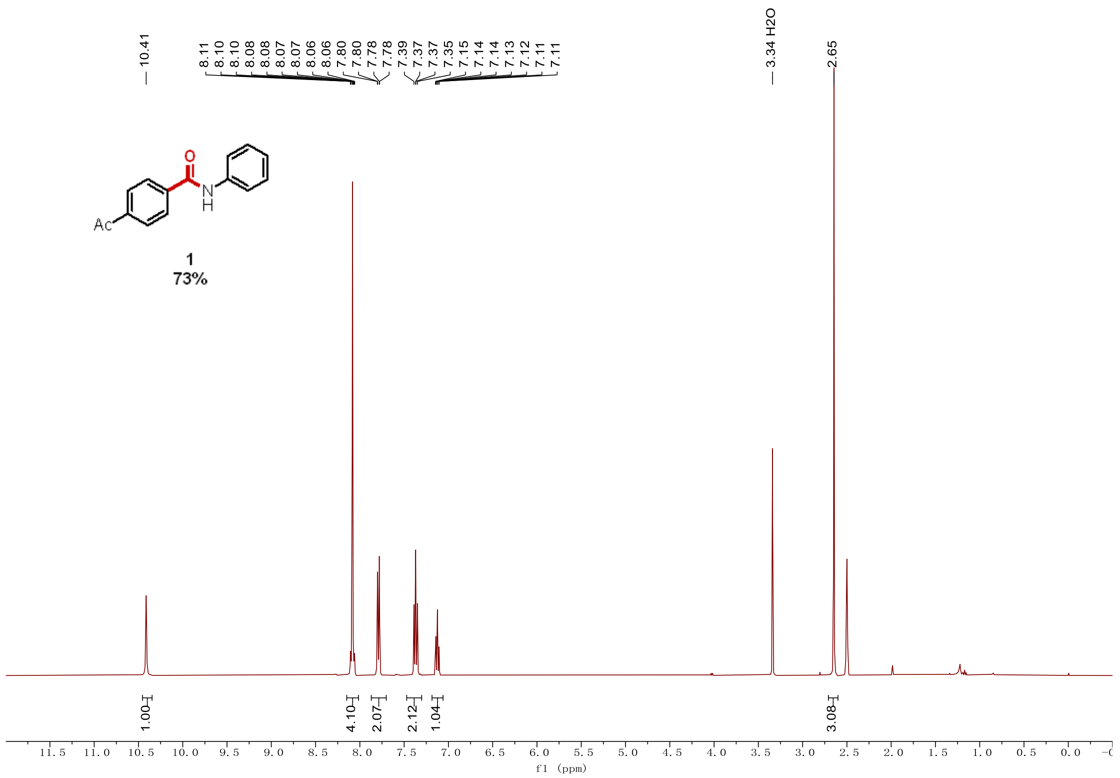

^{1}H NMR spectrum of **1** in DMSO-d_6 (400 MHz)

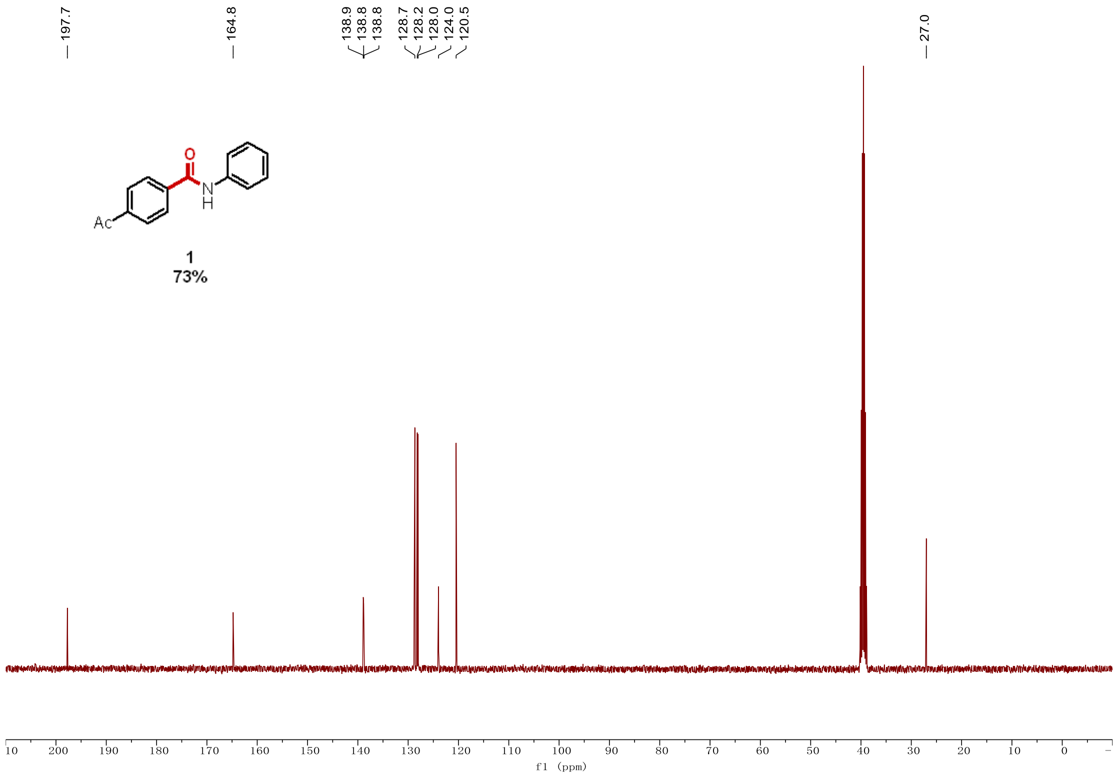

^{13}C NMR spectrum of **1** in DMSO-d_6 (101 MHz)

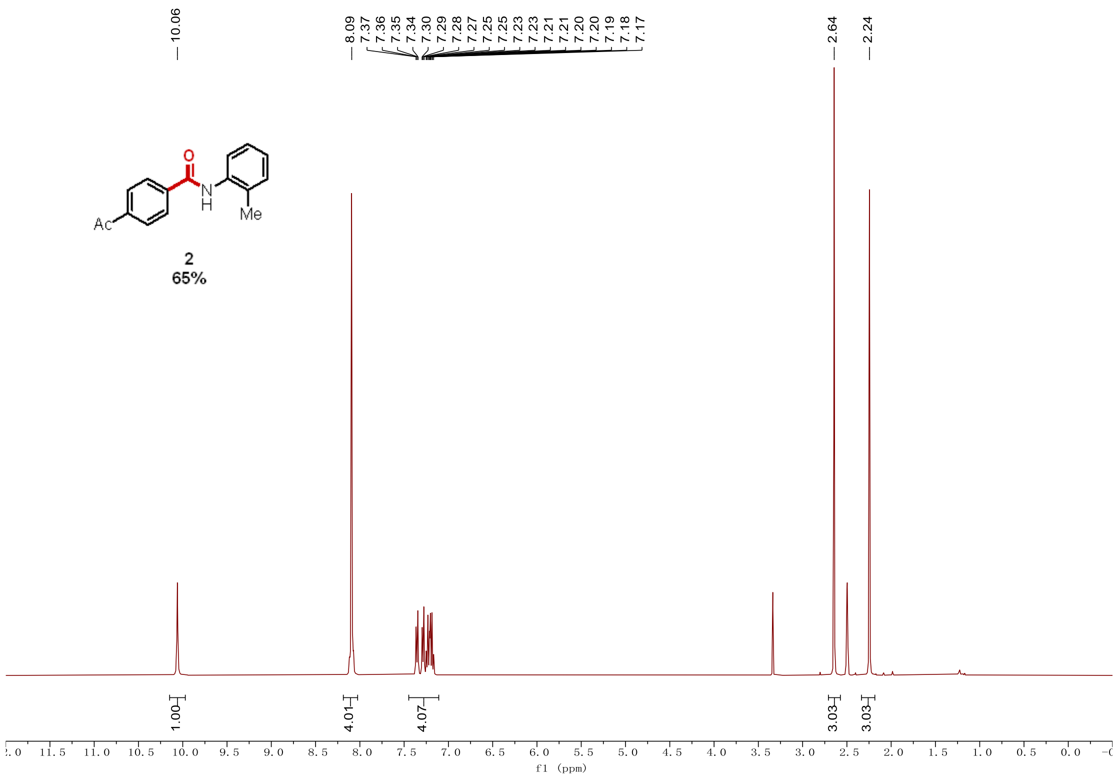

^{1}H NMR spectrum of **2** in DMSO-d_6 (400 MHz)

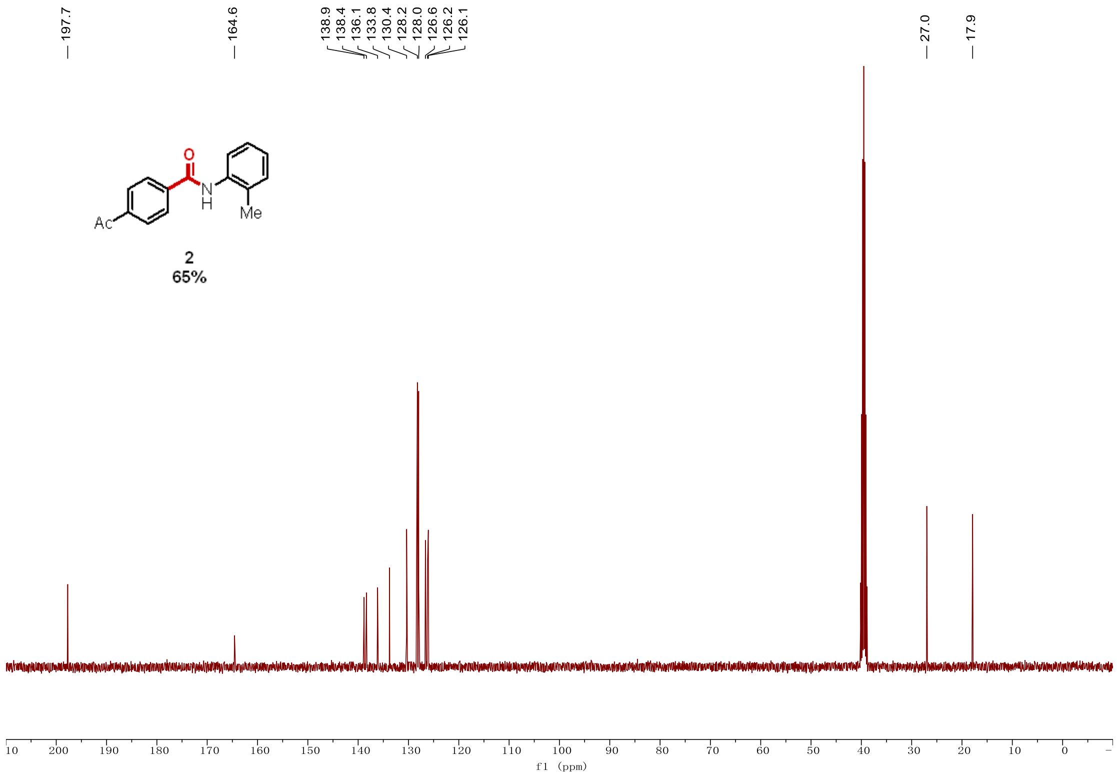

^{13}C NMR spectrum of **2** in DMSO-d_6 (101 MHz)

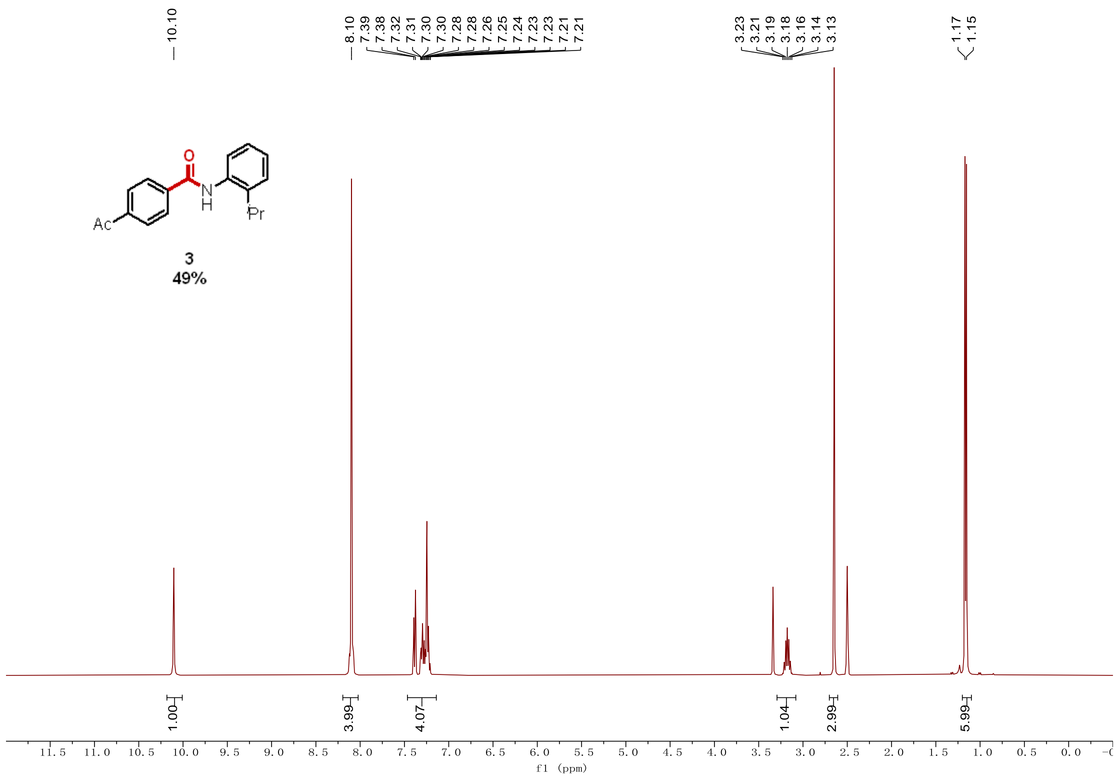

^{1}H NMR spectrum of **3** in DMSO-d_6 (400 MHz)

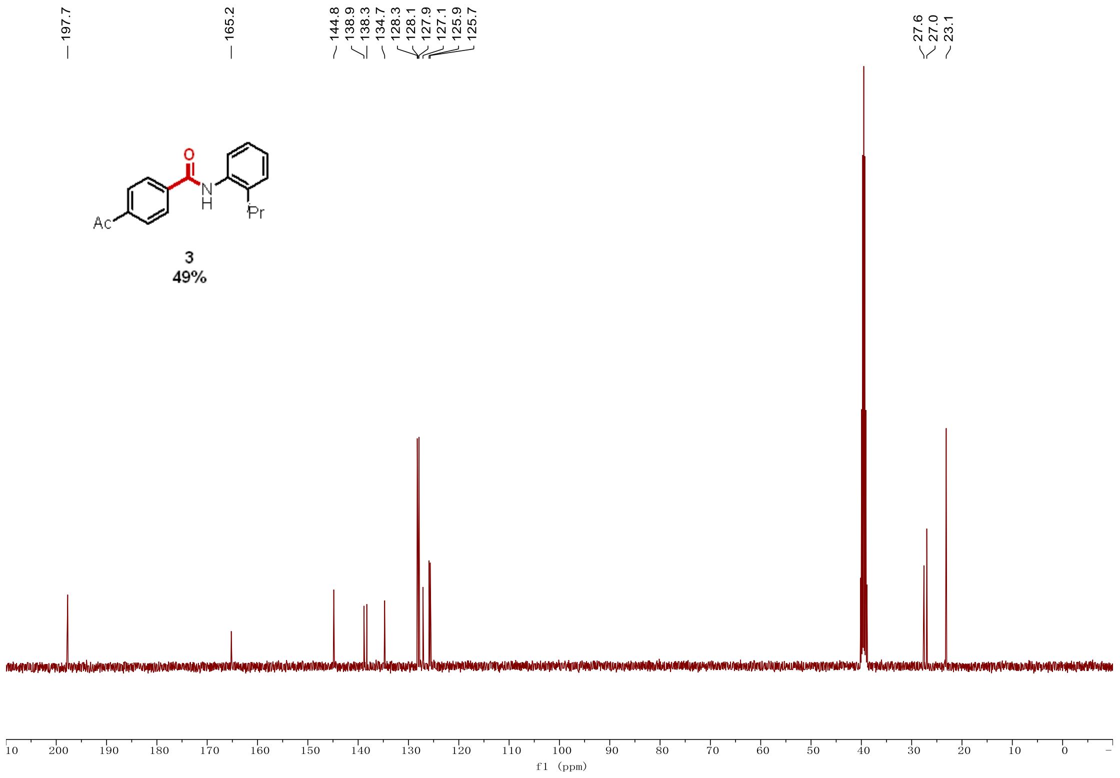

^{13}C NMR spectrum of **3** in DMSO-d_6 (101 MHz)

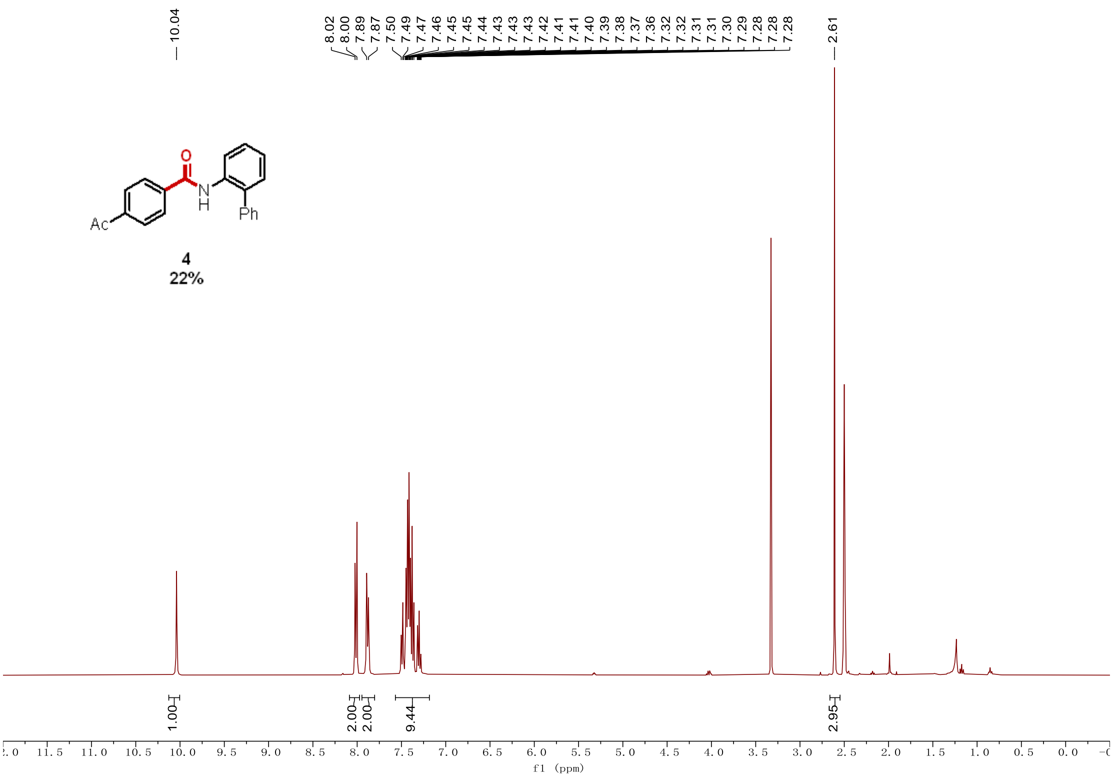

^{1}H NMR spectrum of **4** in DMSO-d_6 (400 MHz)

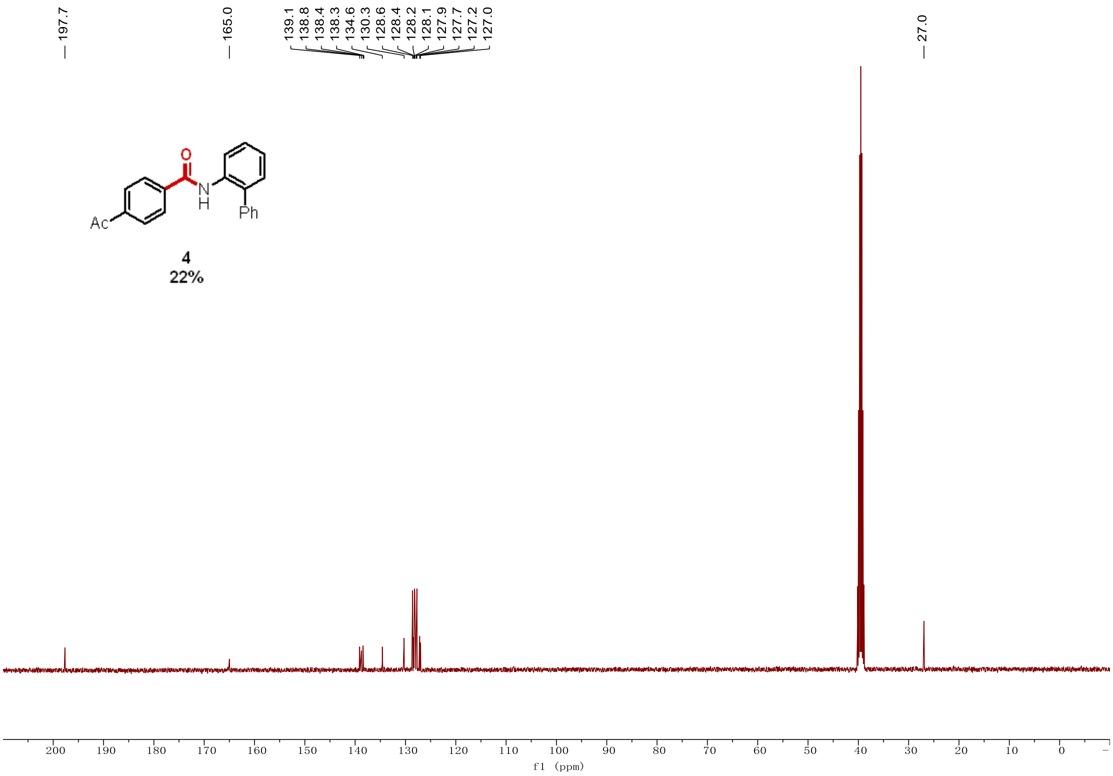

^{13}C NMR spectrum of **4** in DMSO-d_6 (101 MHz)

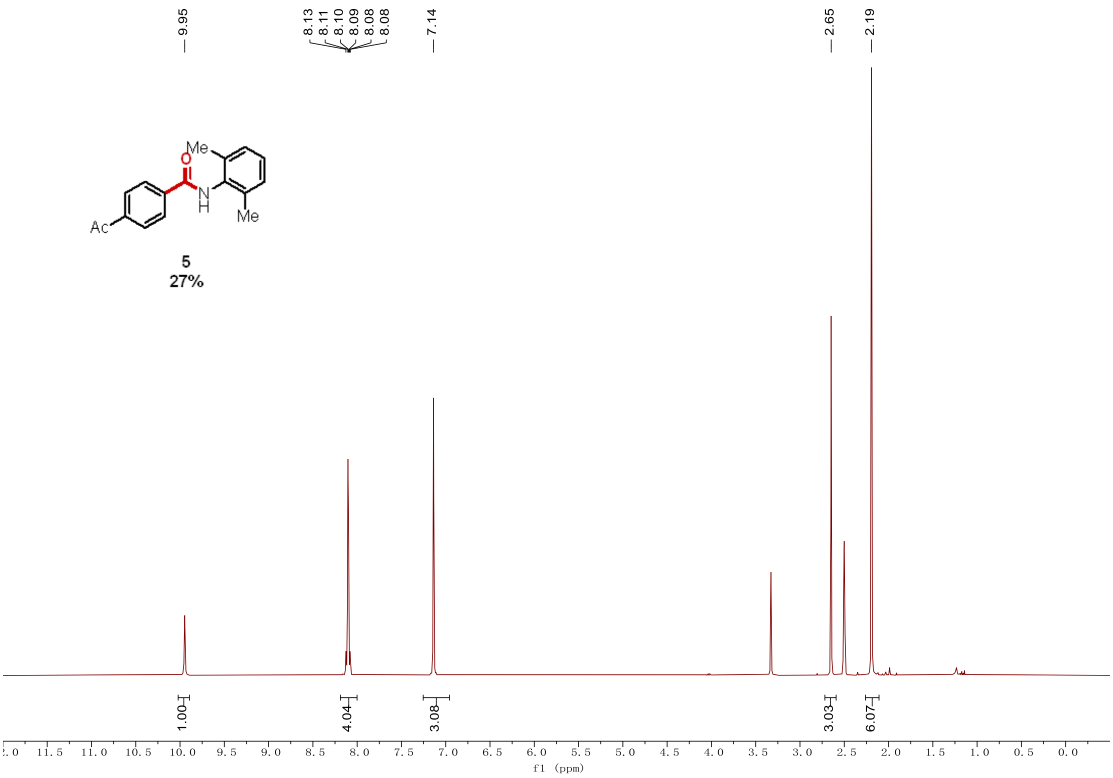

^{1}H NMR spectrum of **5** in DMSO-d_6 (400 MHz)

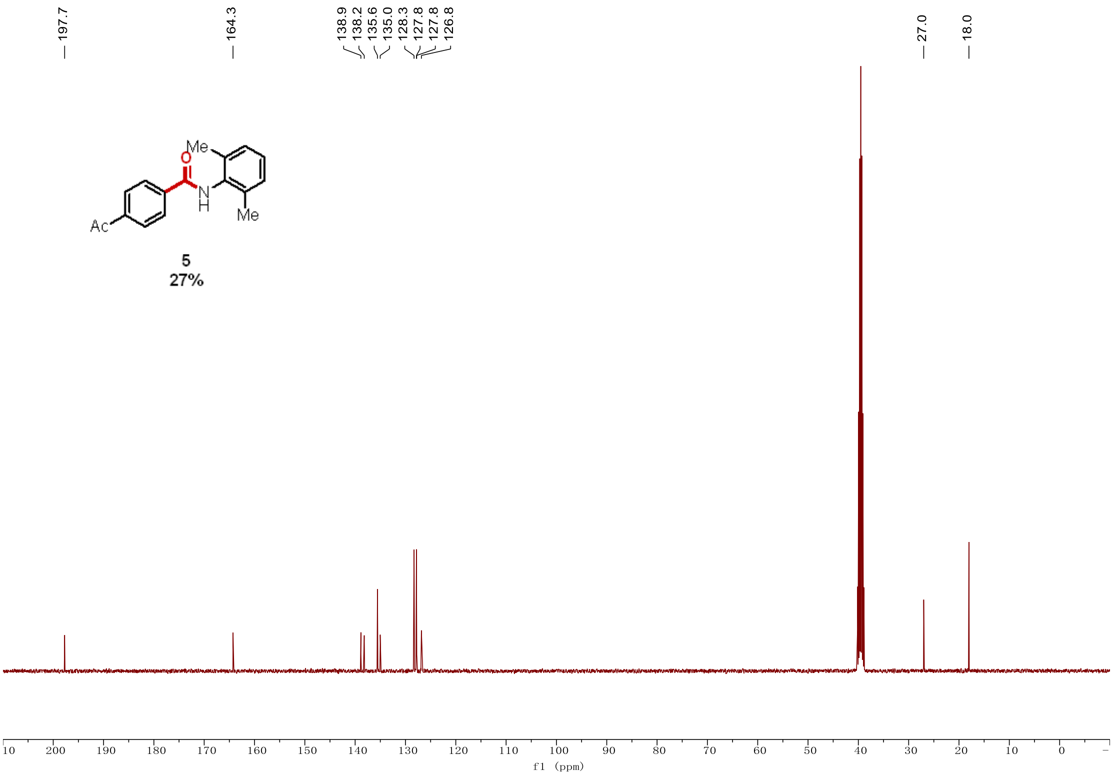

^{13}C NMR spectrum of **5** in DMSO-d_6 (101 MHz)

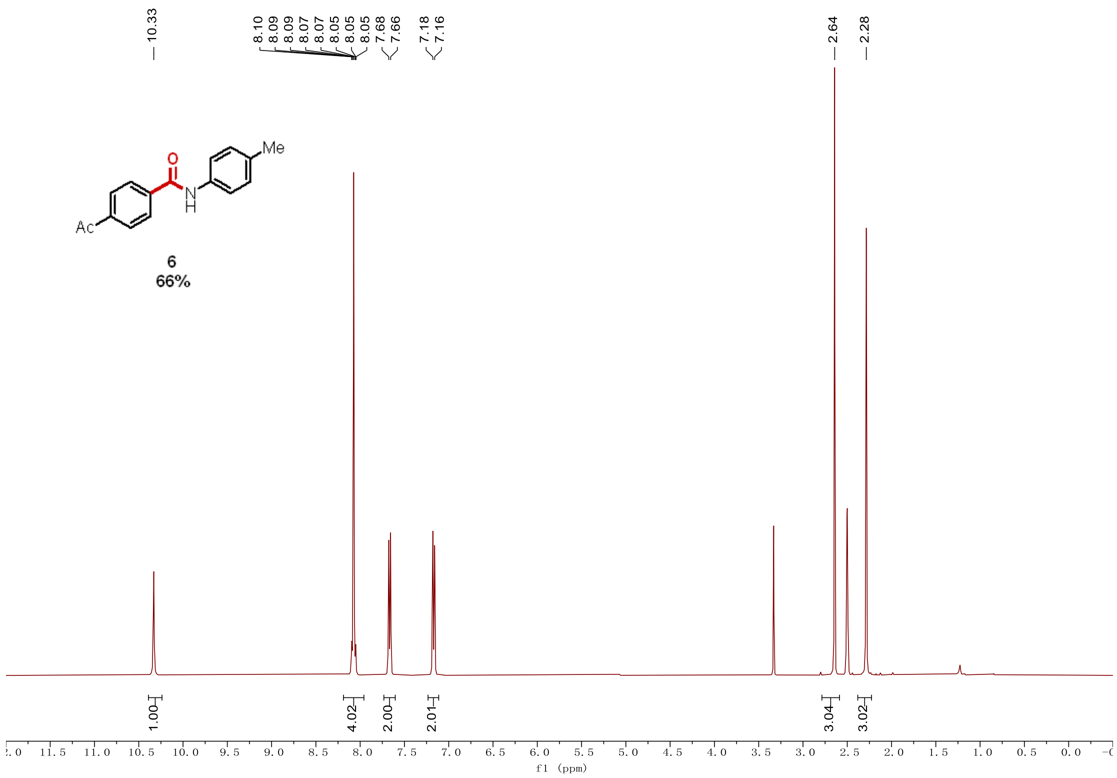

^{1}H NMR spectrum of **6** in DMSO-d_6 (400 MHz)

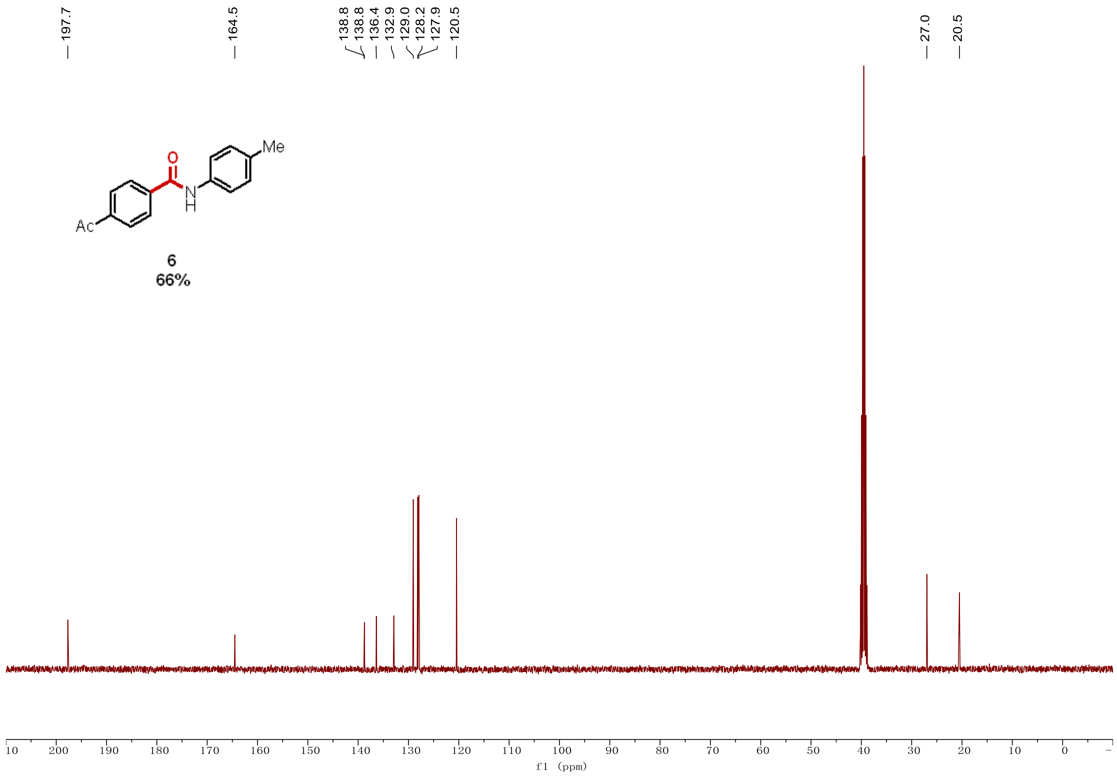

^{13}C NMR spectrum of **6** in DMSO-d_6 (101 MHz)

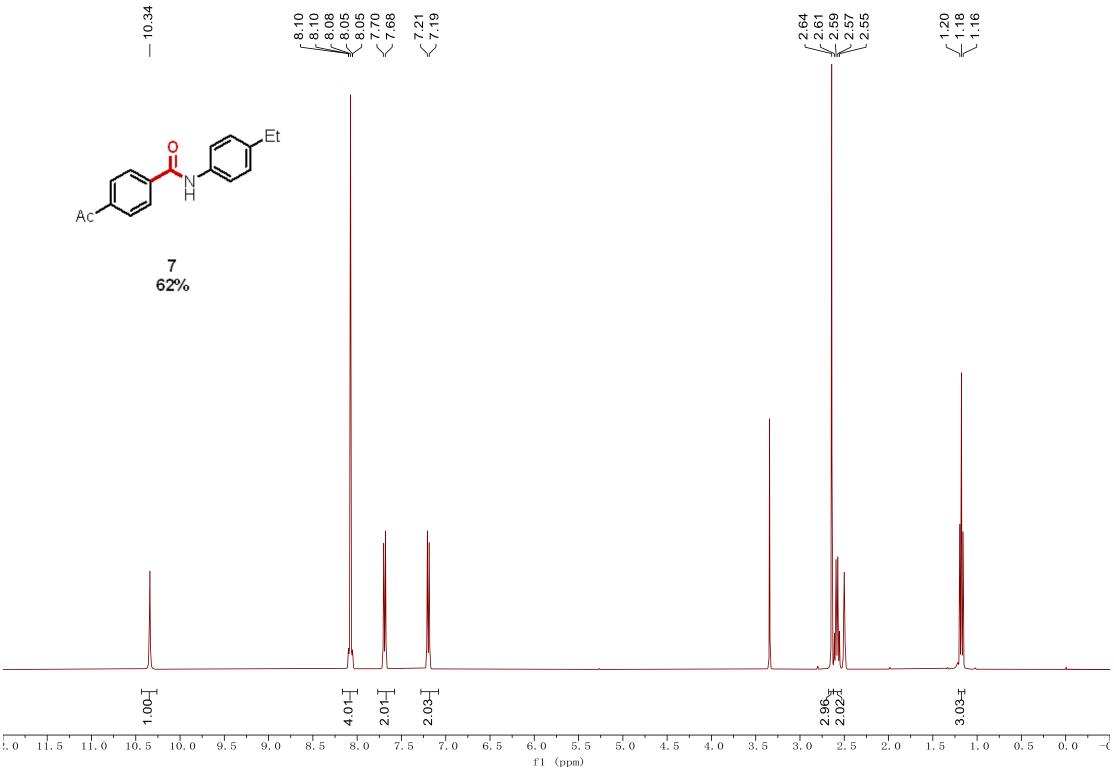

^{1}H NMR spectrum of **7** in DMSO-d_6 (400 MHz)

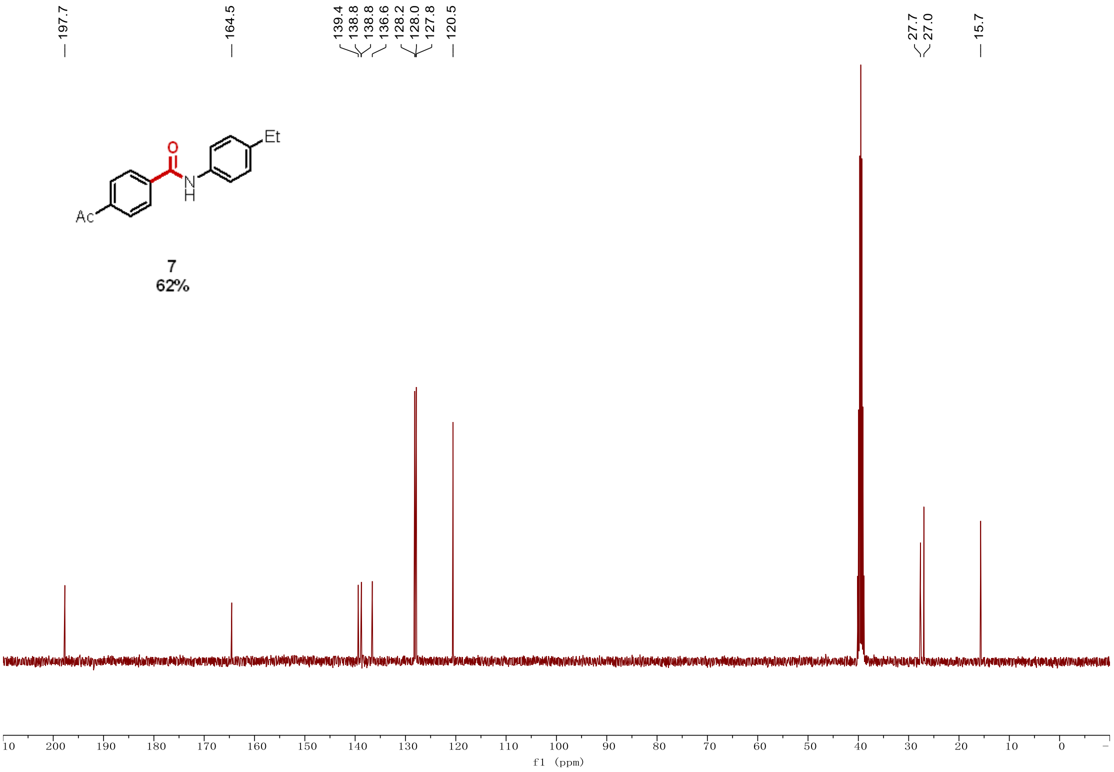

^{13}C NMR spectrum of **7** in DMSO-d_6 (101 MHz)

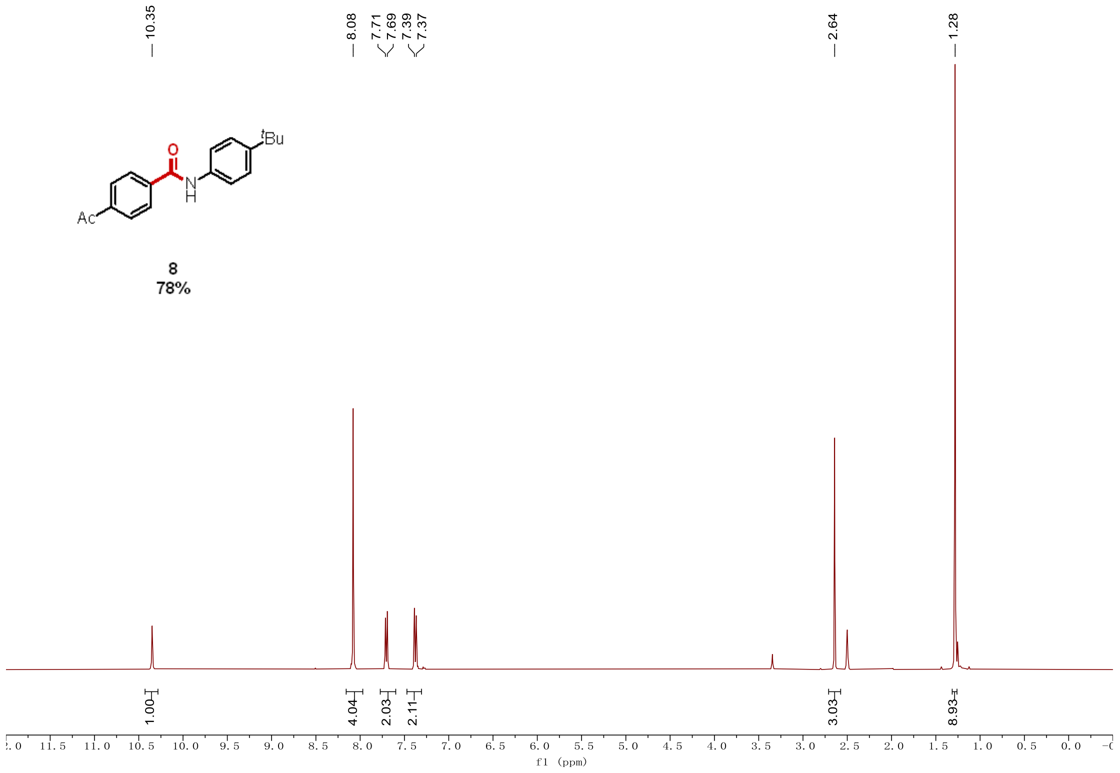

^{1}H NMR spectrum of **8** in DMSO-d_6 (400 MHz)

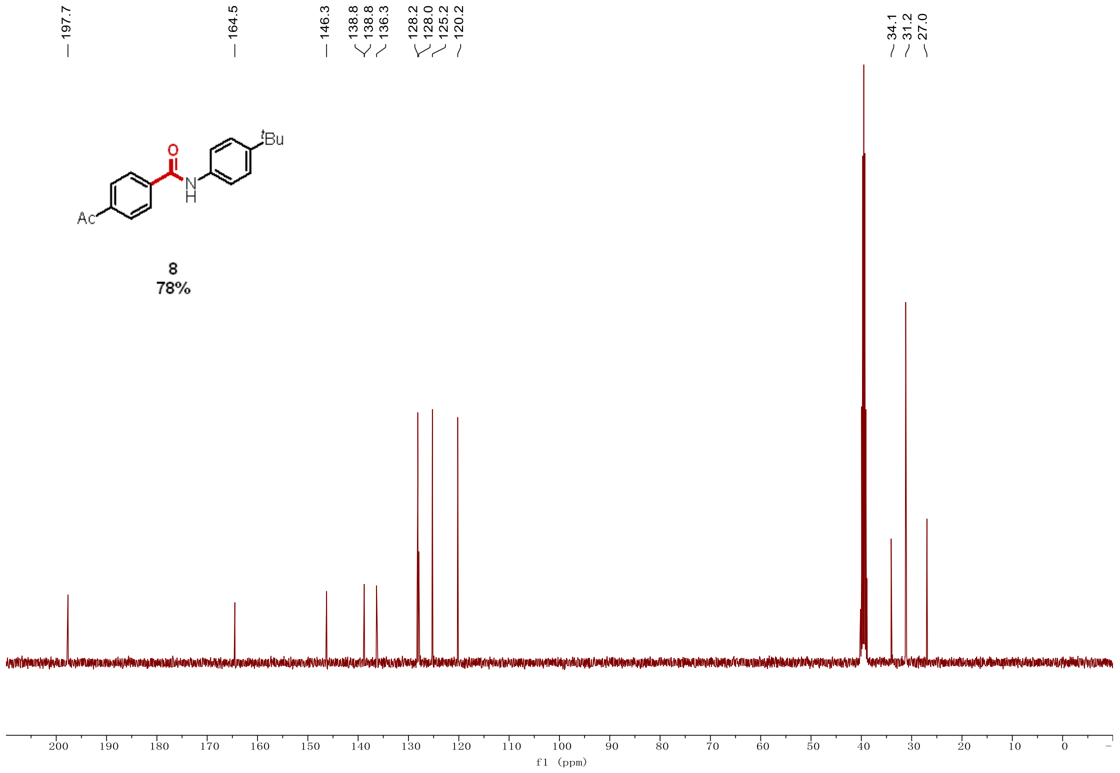

^{13}C NMR spectrum of **8** in DMSO-d_6 (101 MHz)

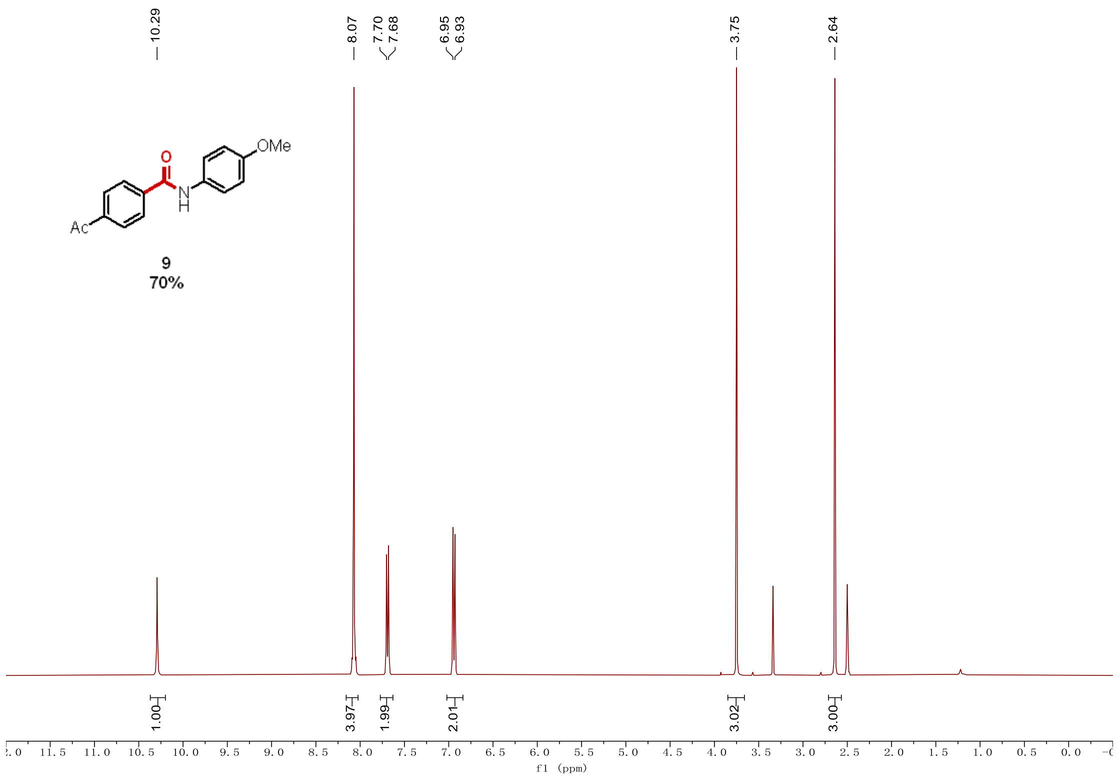

^{1}H NMR spectrum of **9** in DMSO-d_6 (400 MHz)

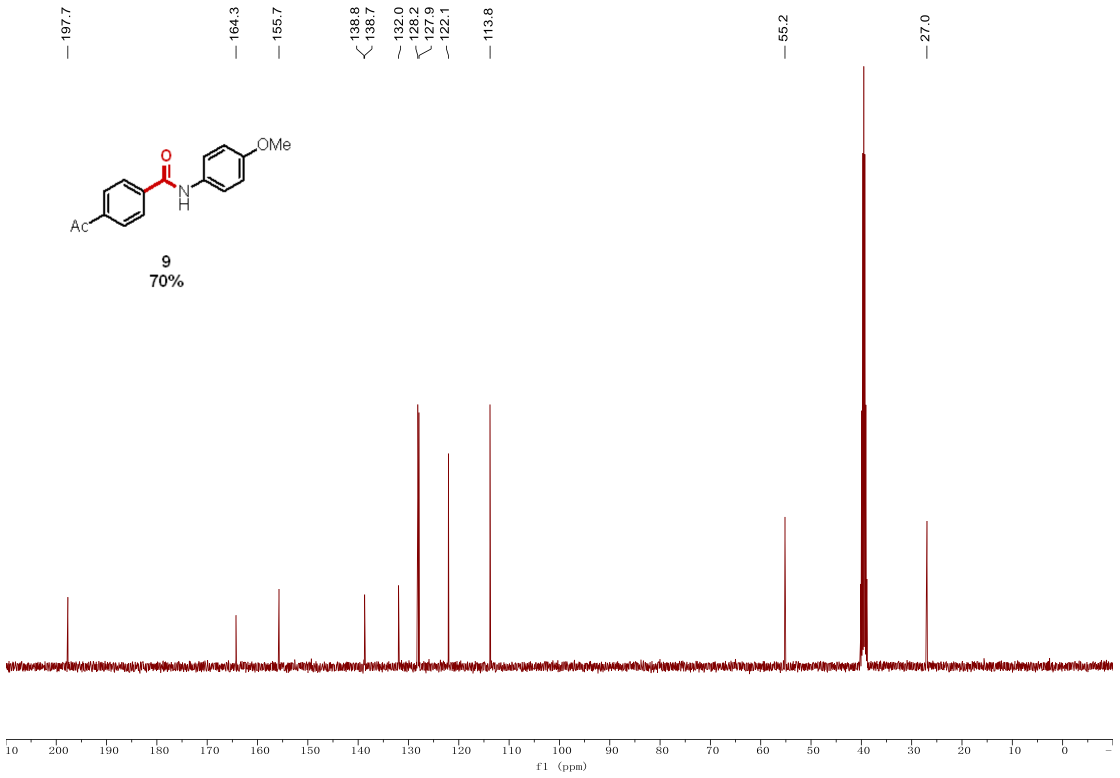

^{13}C NMR spectrum of **9** in DMSO-d_6 (101 MHz)

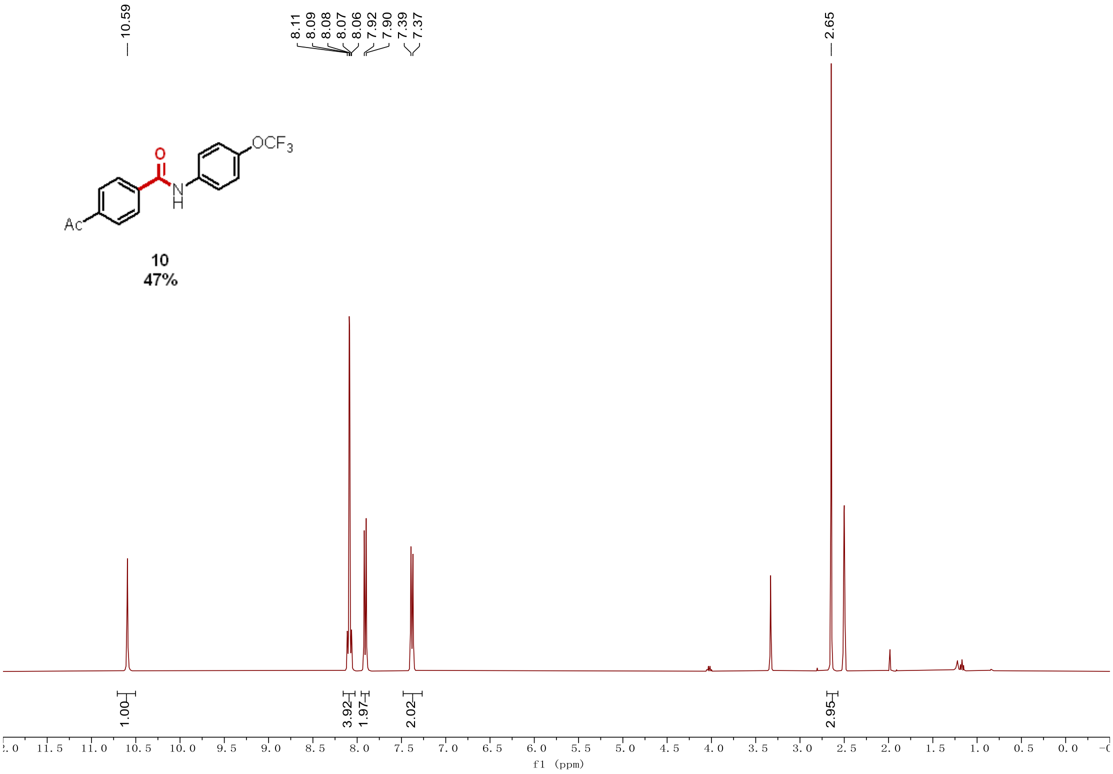

^{1}H NMR spectrum of **10** in DMSO-d_6 (400 MHz)

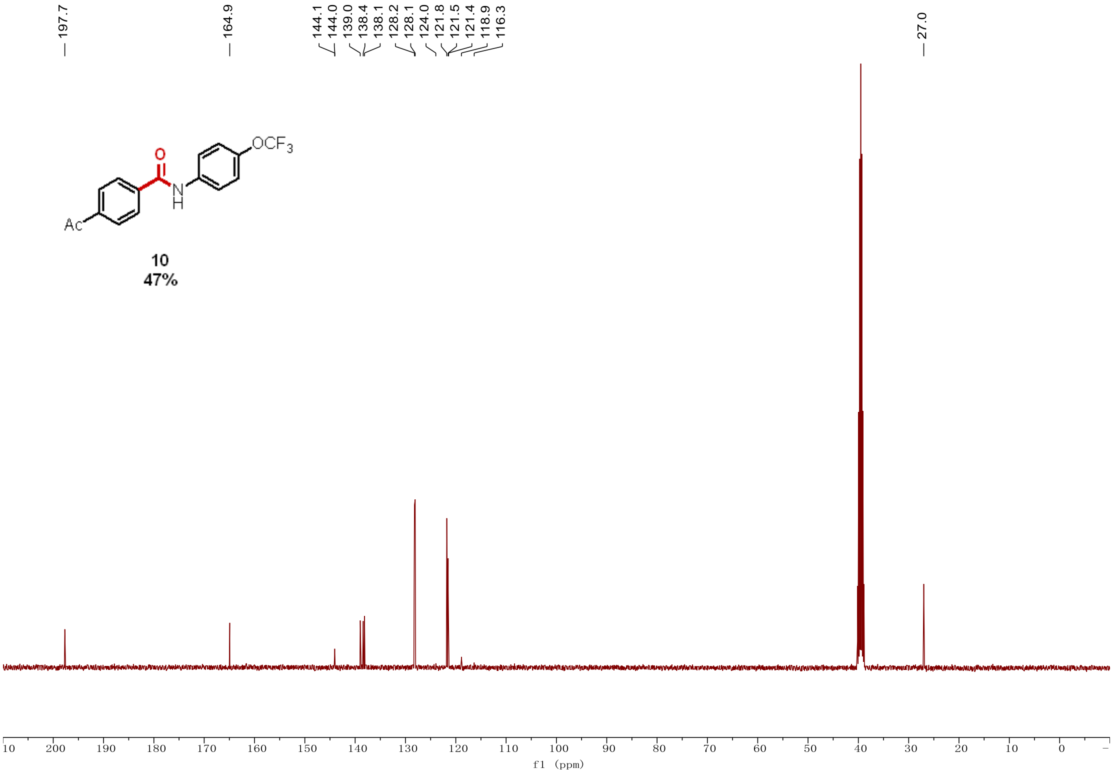

^{13}C NMR spectrum of **10** in DMSO-d_6 (101 MHz)

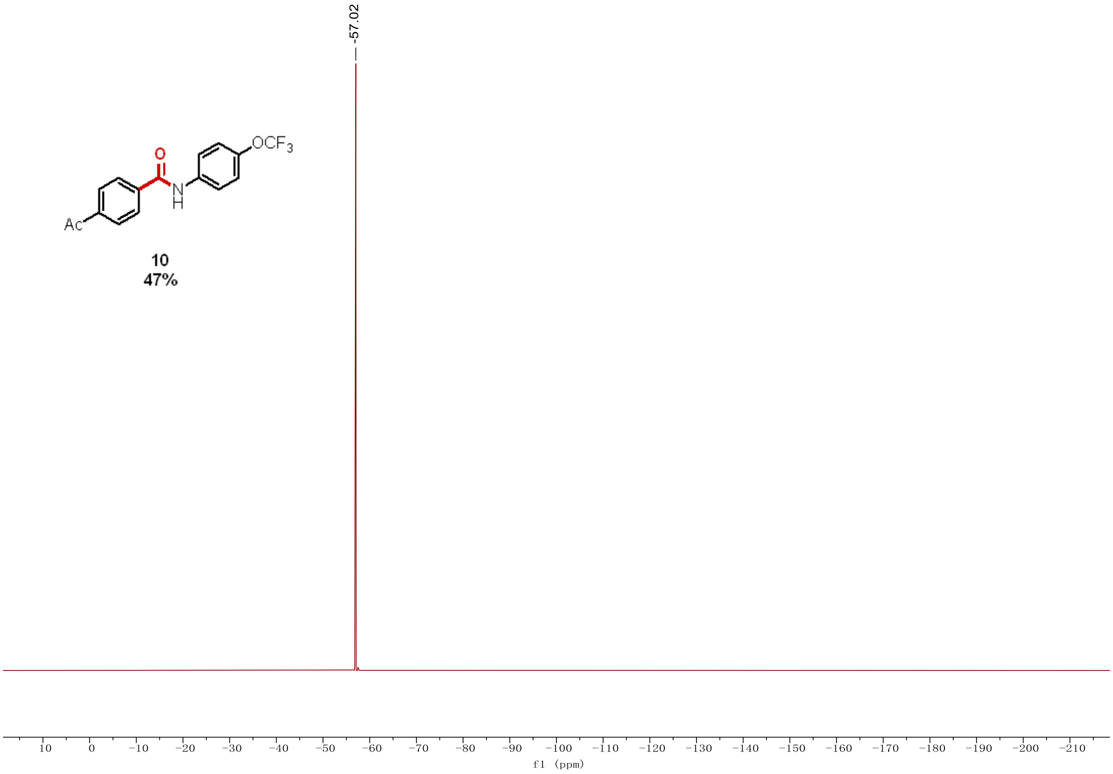

^{19}F NMR spectrum of **10** in DMSO-d_6 (376 MHz)

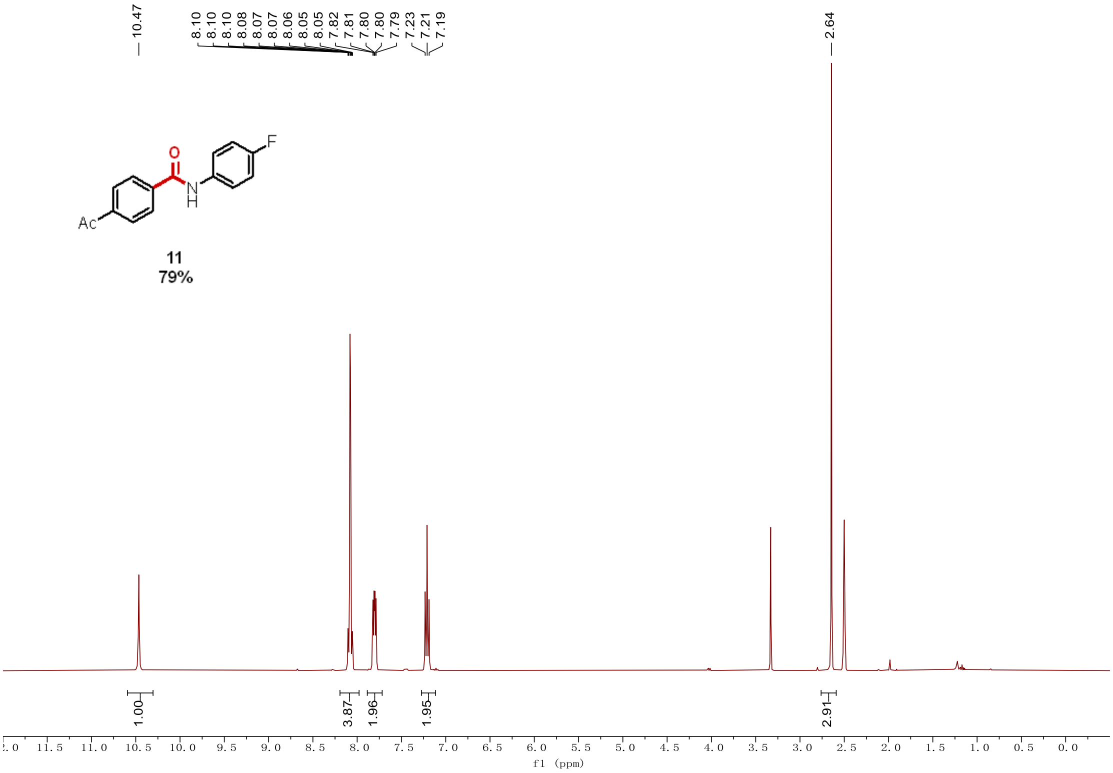

^{1}H NMR spectrum of **11** in DMSO-d_6 (400 MHz)

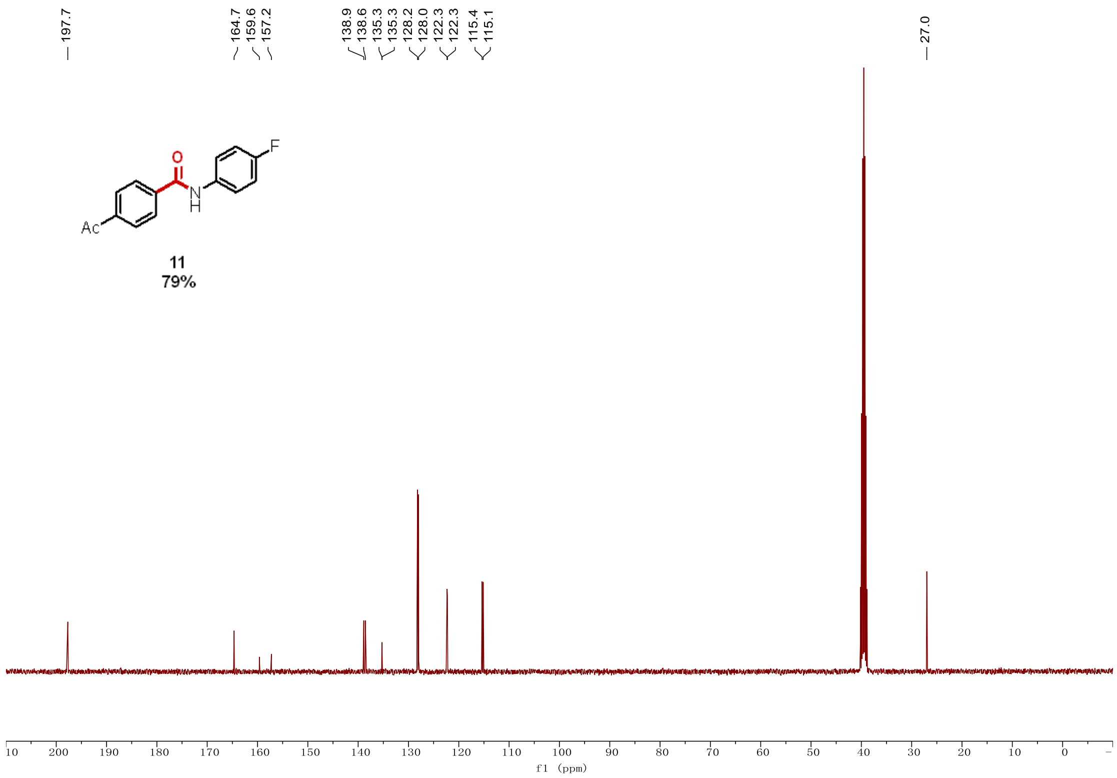

^{13}C NMR spectrum of **11** in DMSO-d_6 (101 MHz)

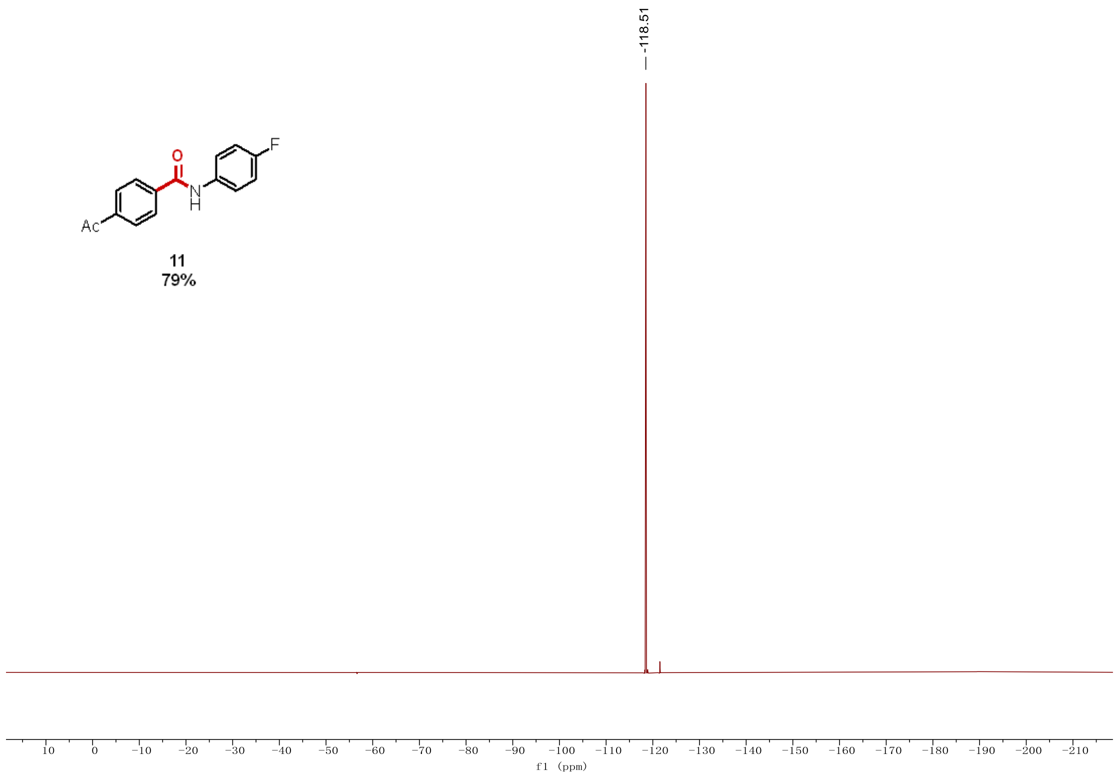

^{19}F NMR spectrum of **11** in DMSO-d_6 (376 MHz)

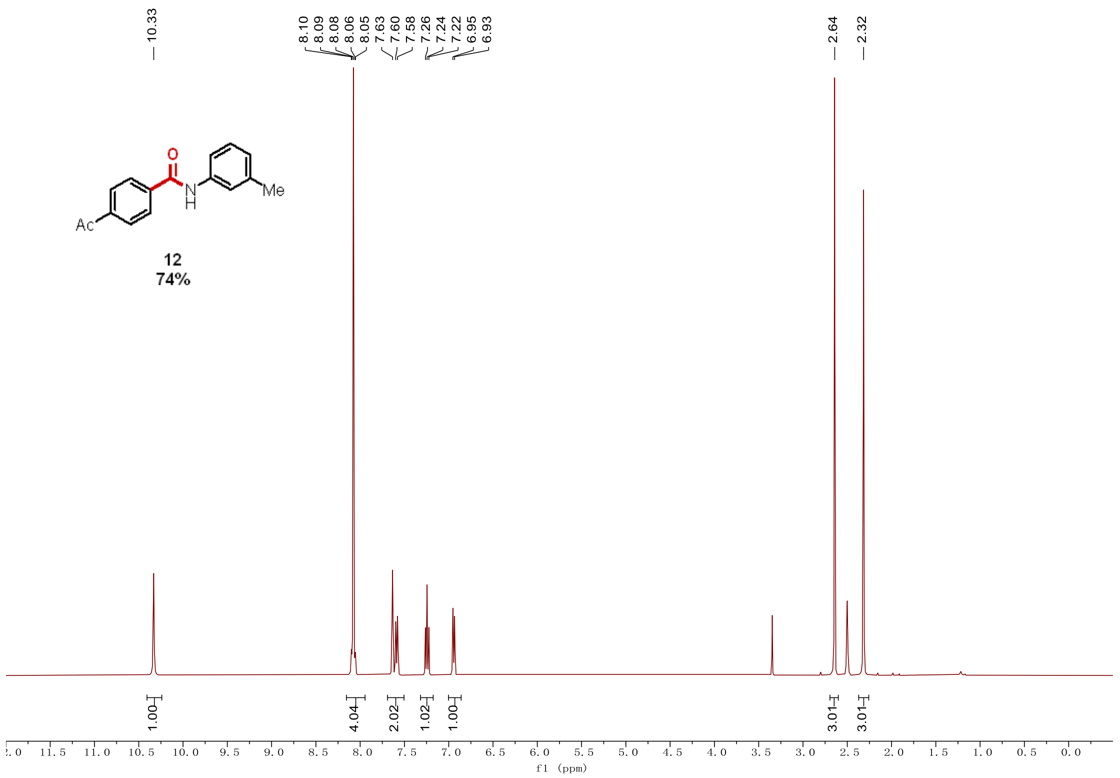

^{1}H NMR spectrum of **12** in DMSO-d_6 (400 MHz)

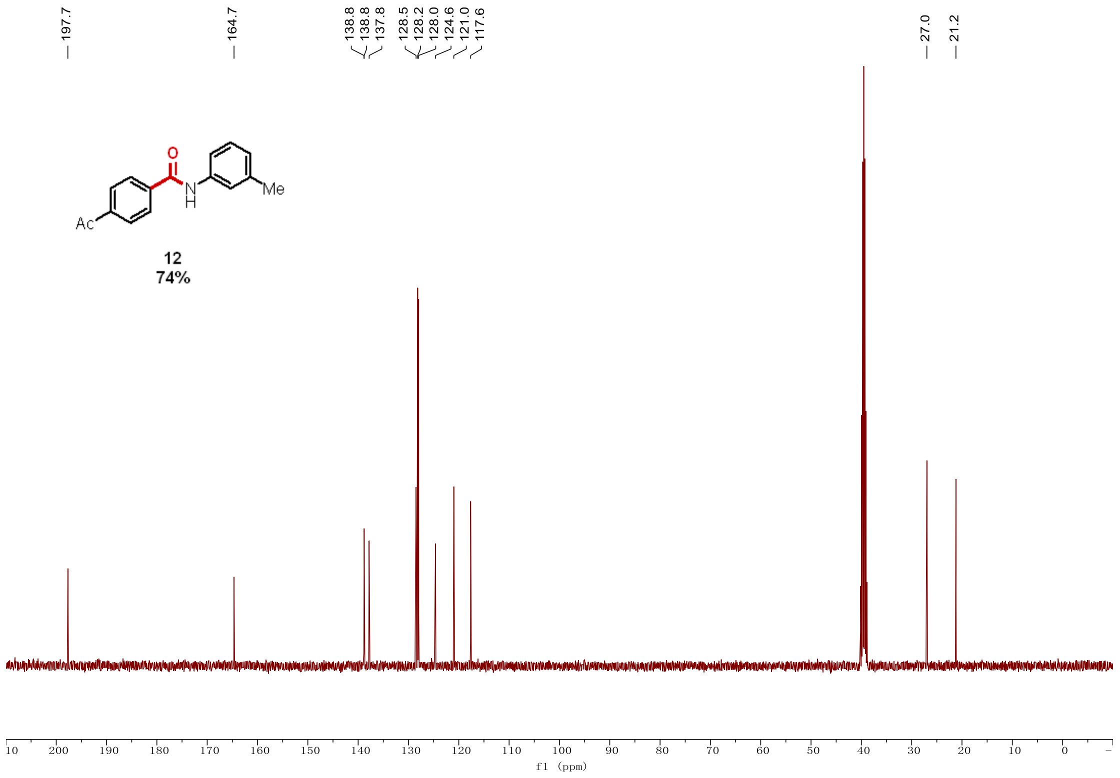

^{13}C NMR spectrum of **12** in DMSO-d_6 (101 MHz)

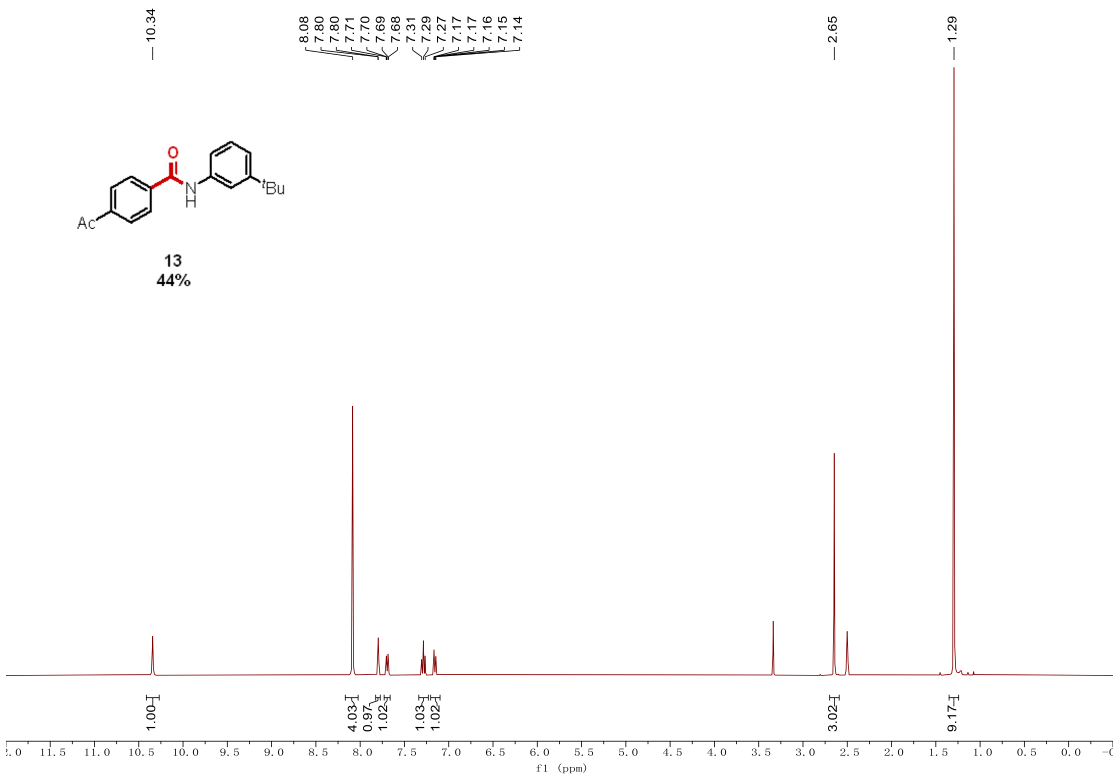

^{1}H NMR spectrum of **13** in DMSO-d_6 (400 MHz)

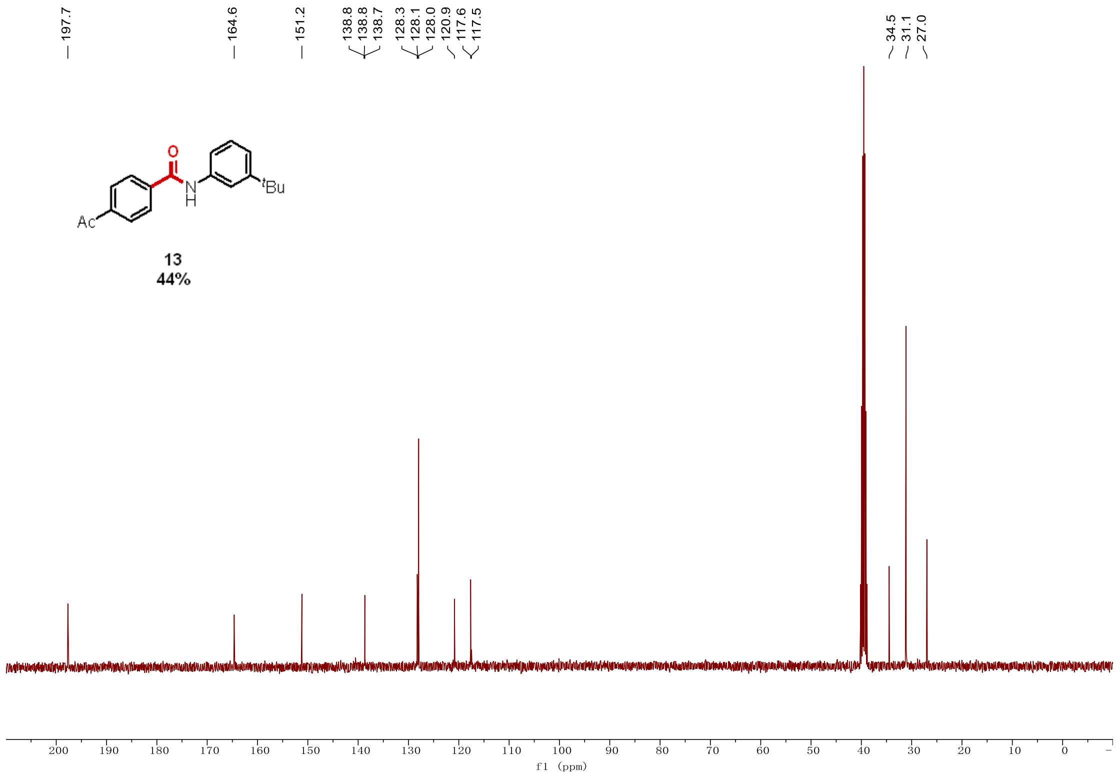

^{13}C NMR spectrum of **13** in DMSO-d_6 (101 MHz)

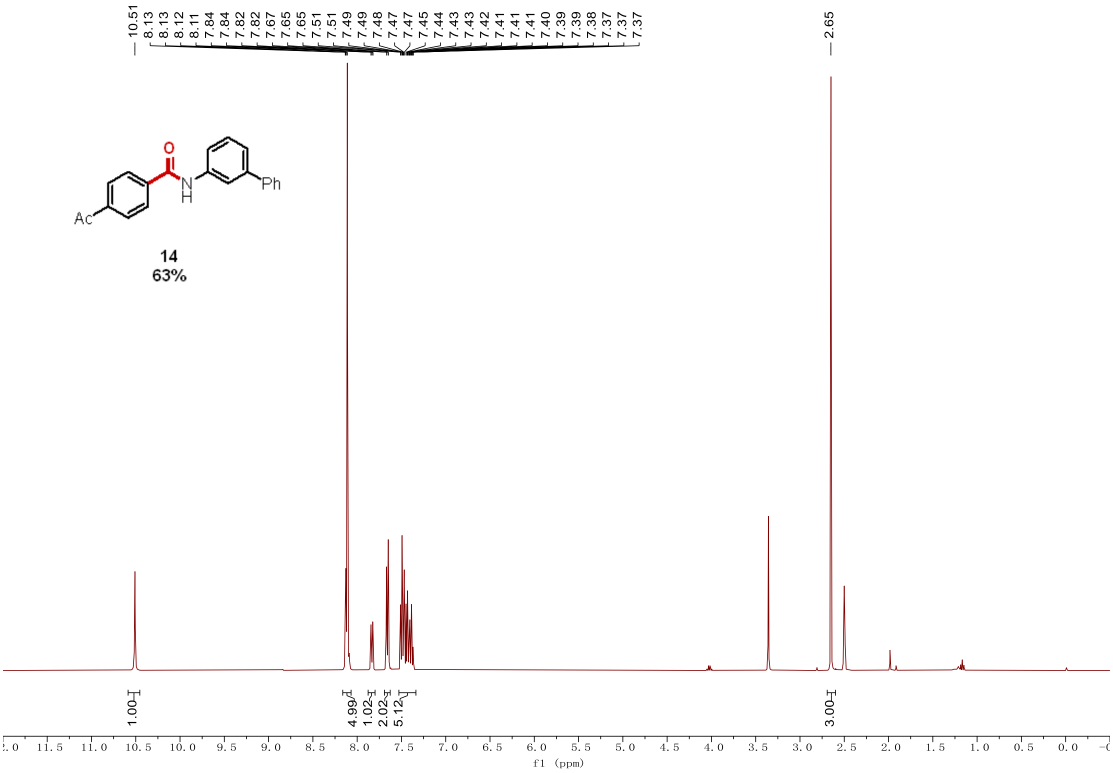

^{1}H NMR spectrum of **14** in DMSO-d_6 (400 MHz)

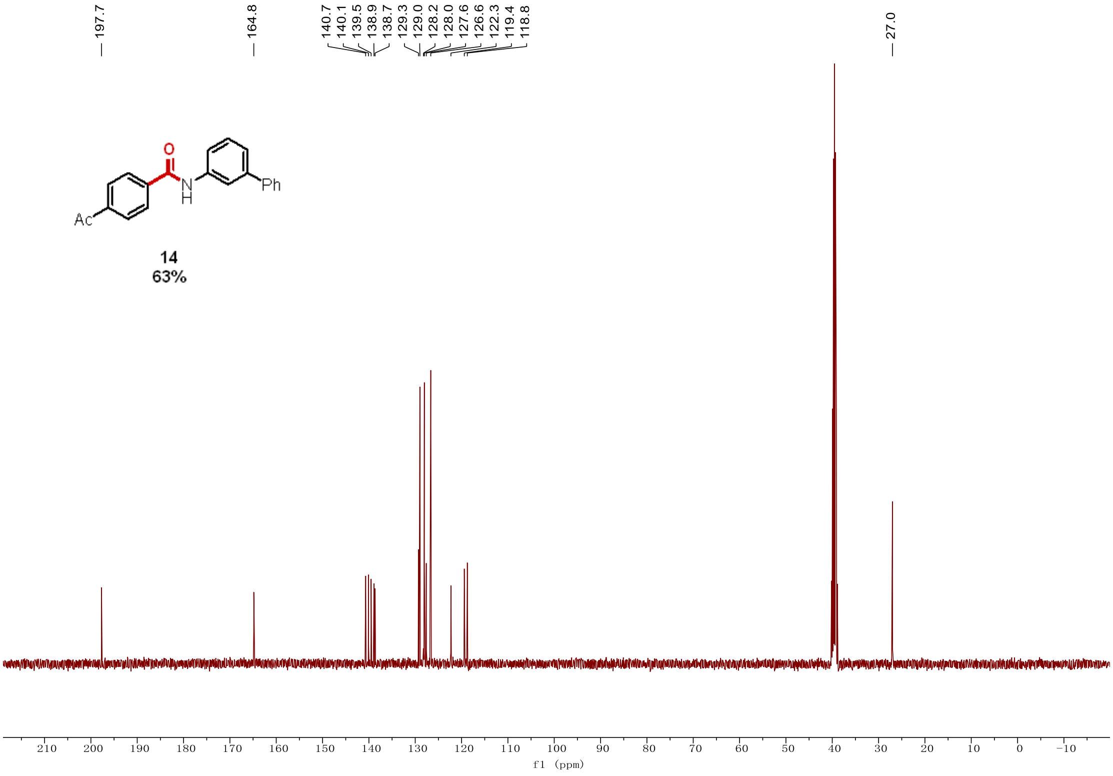

^{13}C NMR spectrum of **14** in DMSO-d_6 (101 MHz)

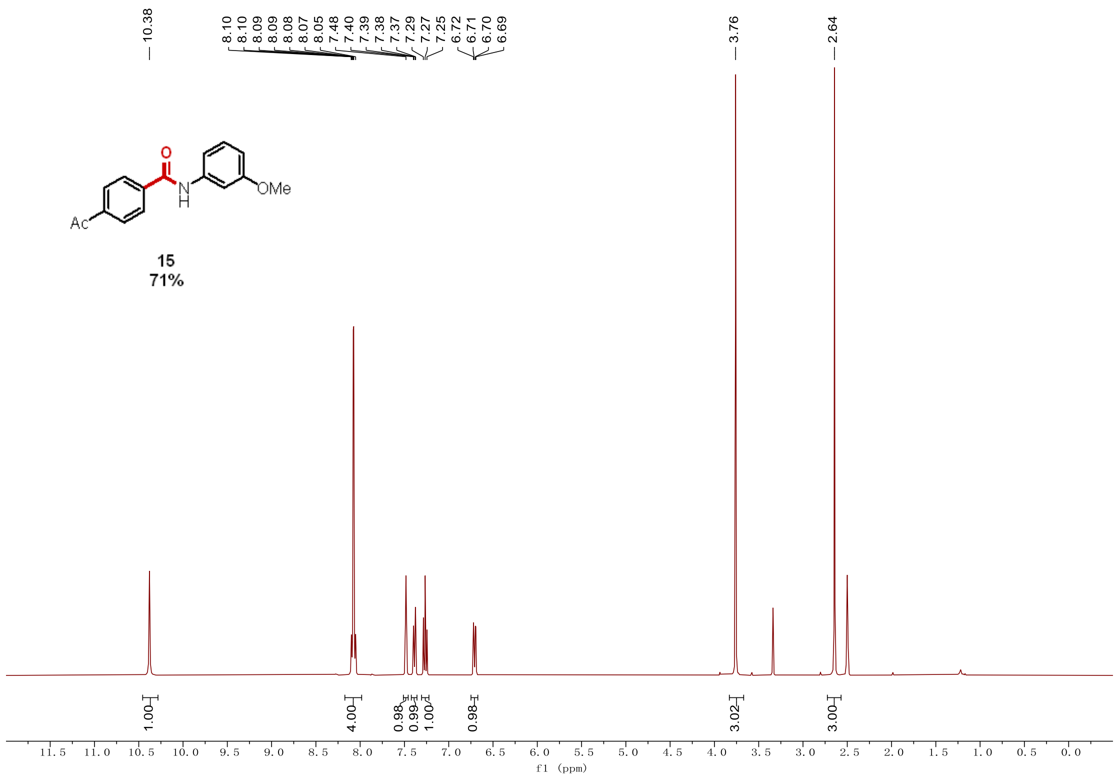

^{1}H NMR spectrum of **15** in DMSO-d_6 (400 MHz)

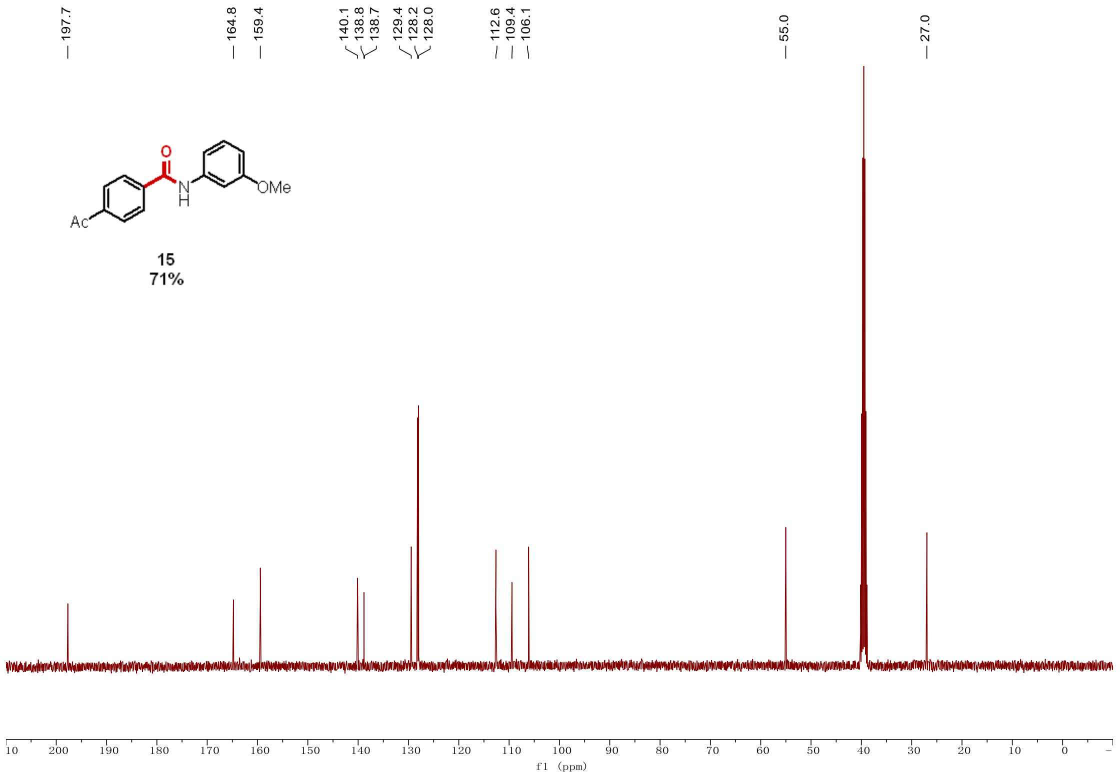

^{13}C NMR spectrum of **15** in DMSO-d_6 (101 MHz)

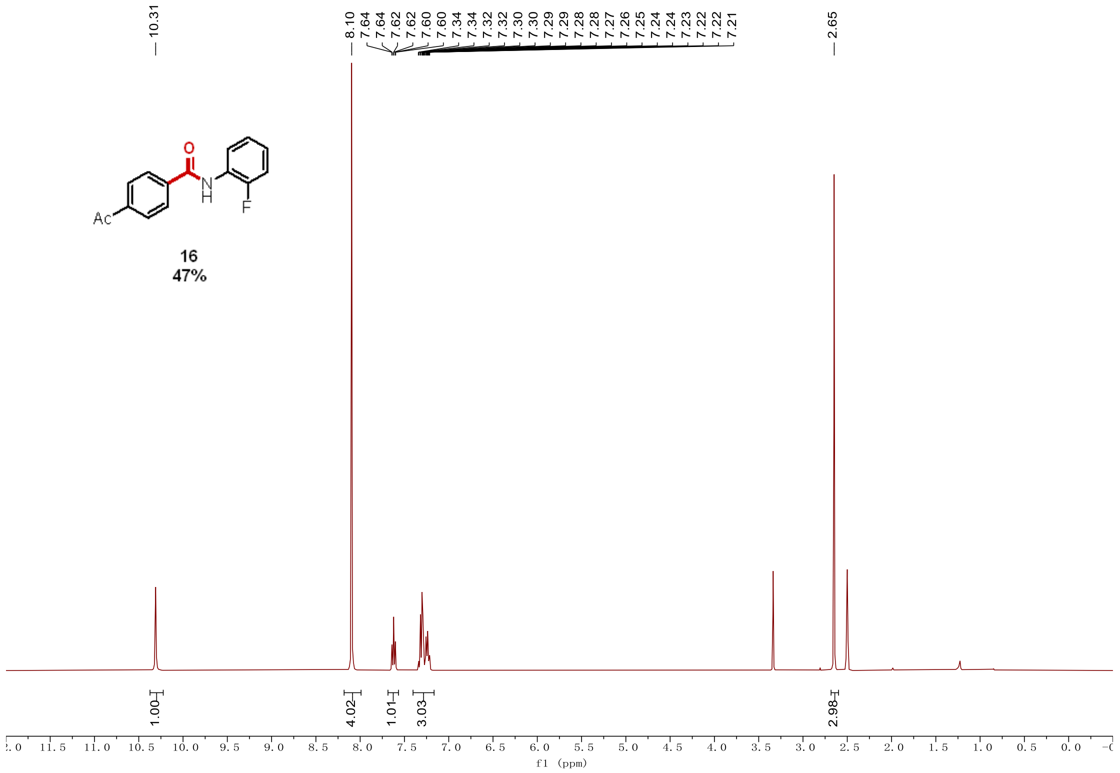

^{1}H NMR spectrum of **16** in DMSO-d_6 (400 MHz)

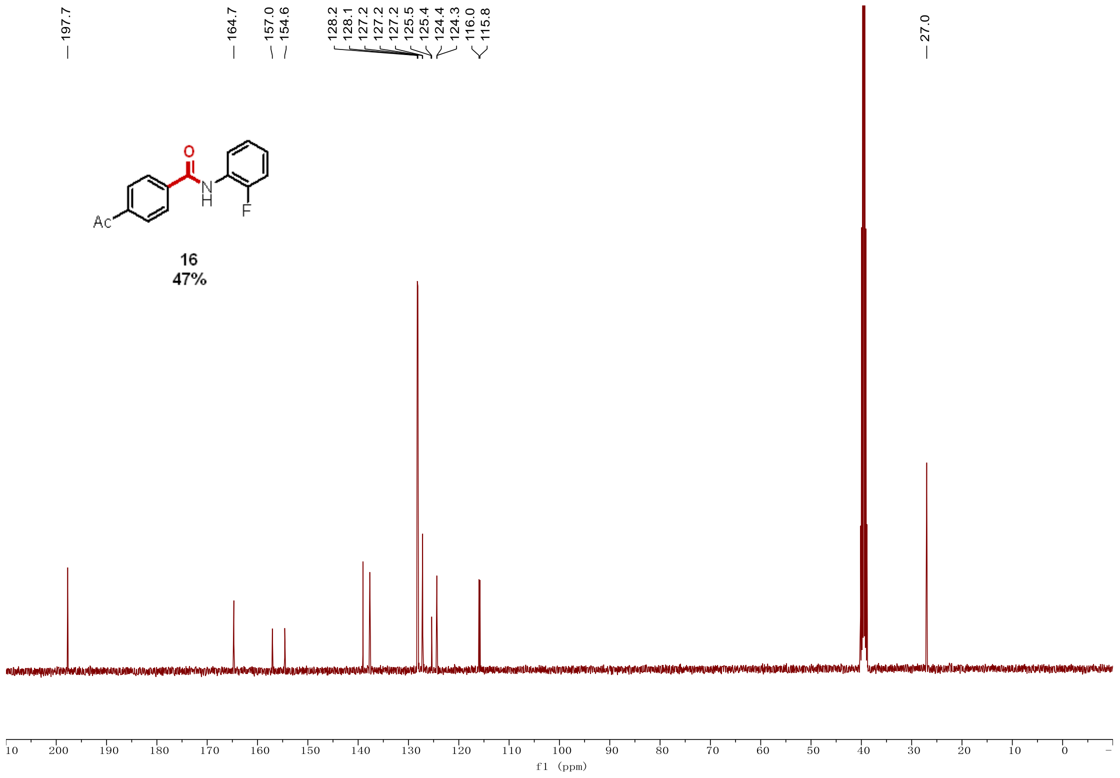

^{13}C NMR spectrum of **16** in DMSO-d_6 (101 MHz)

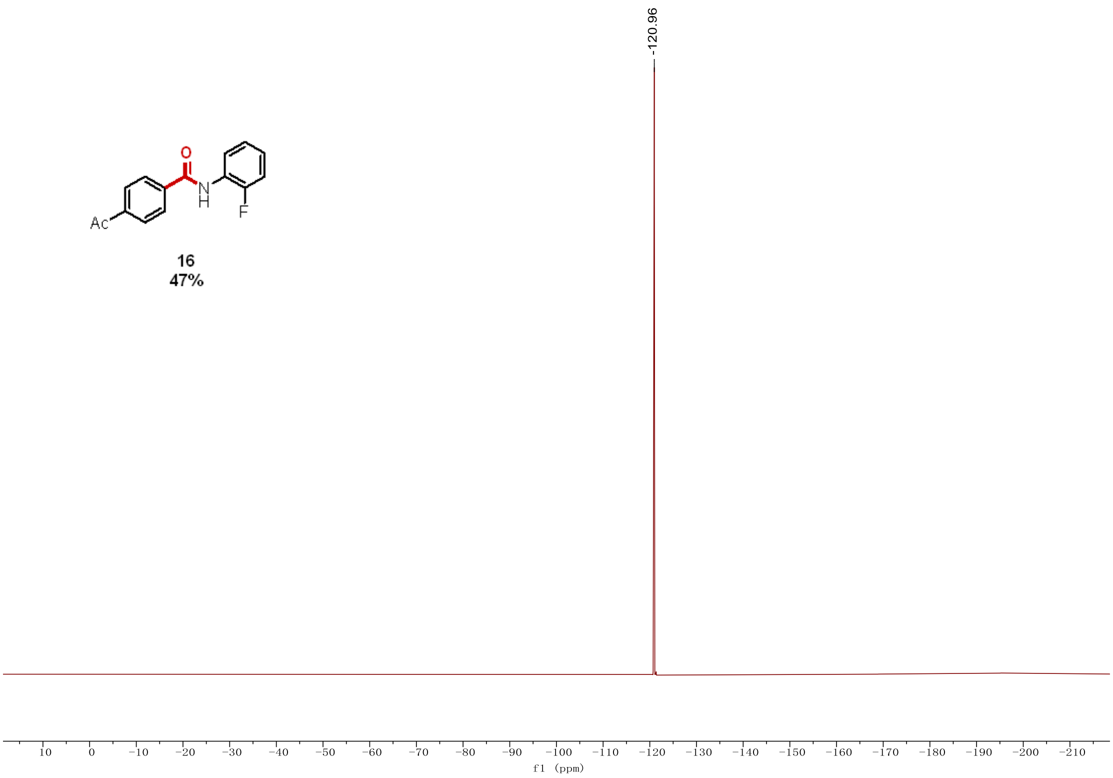

^{19}F NMR spectrum of **16** in DMSO-d_6 (376 MHz)

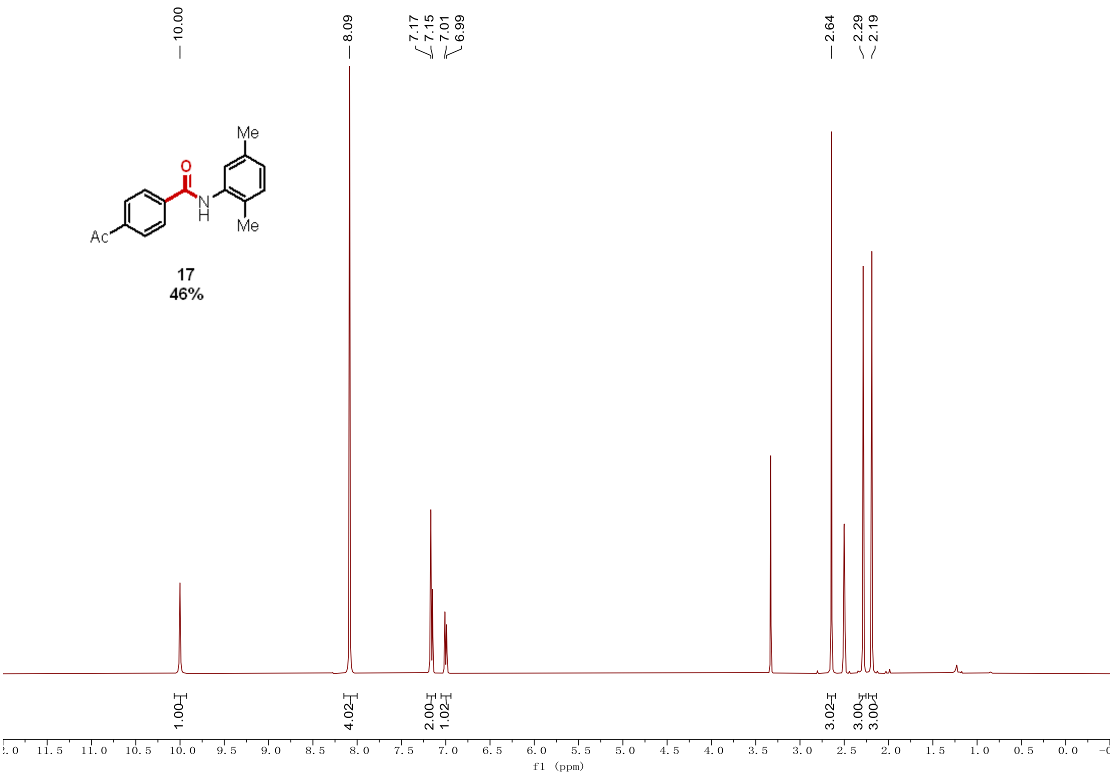

^{1}H NMR spectrum of **17** in DMSO-d_6 (400 MHz)

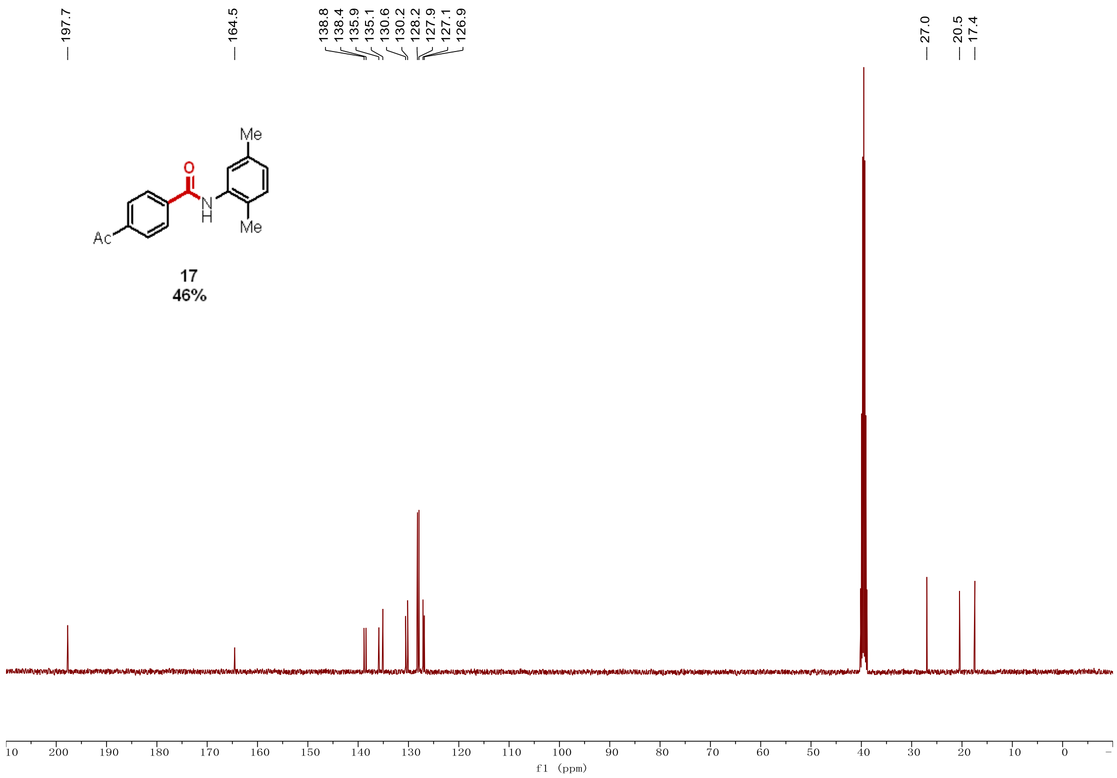

^{13}C NMR spectrum of **17** in DMSO-d_6 (101 MHz)

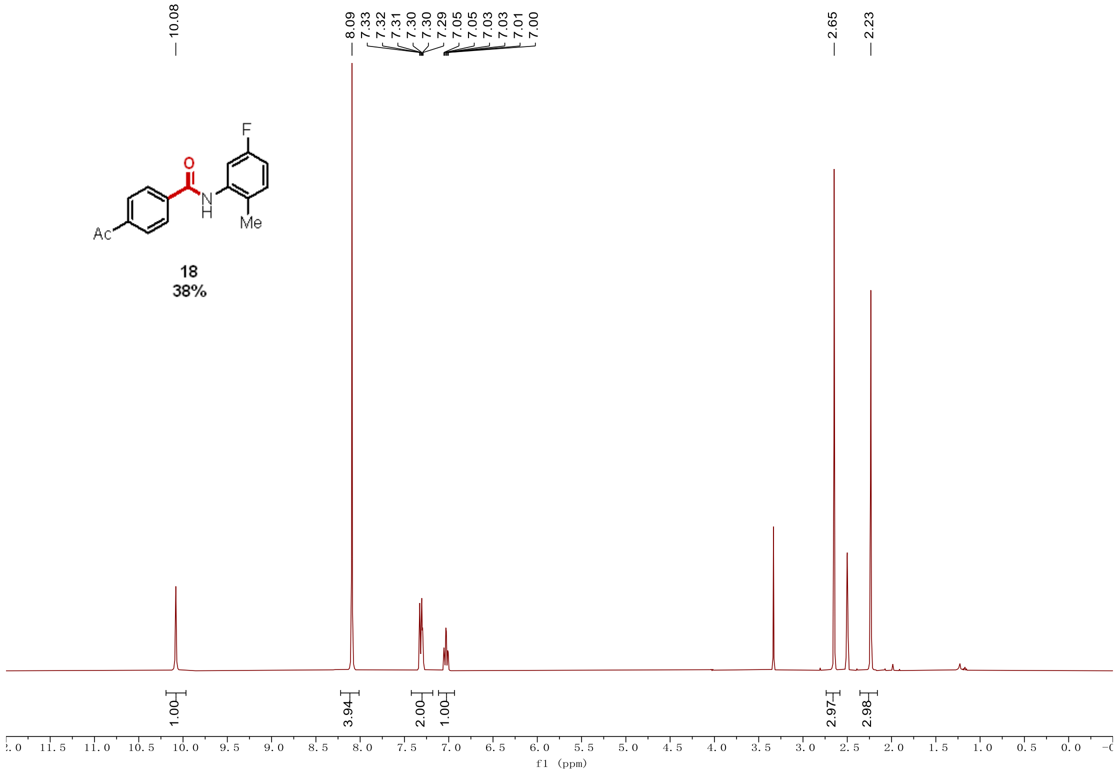

^{1}H NMR spectrum of **18** in DMSO-d_6 (400 MHz)

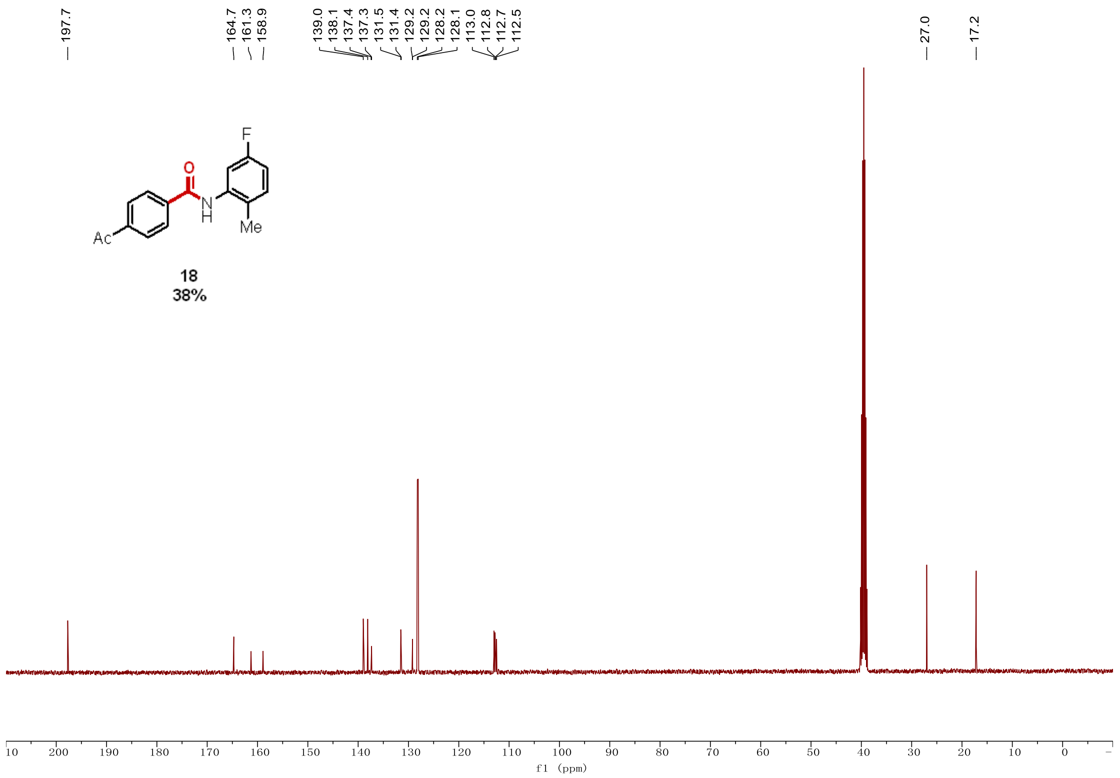

^{13}C NMR spectrum of **18** in DMSO-d_6 (101 MHz)

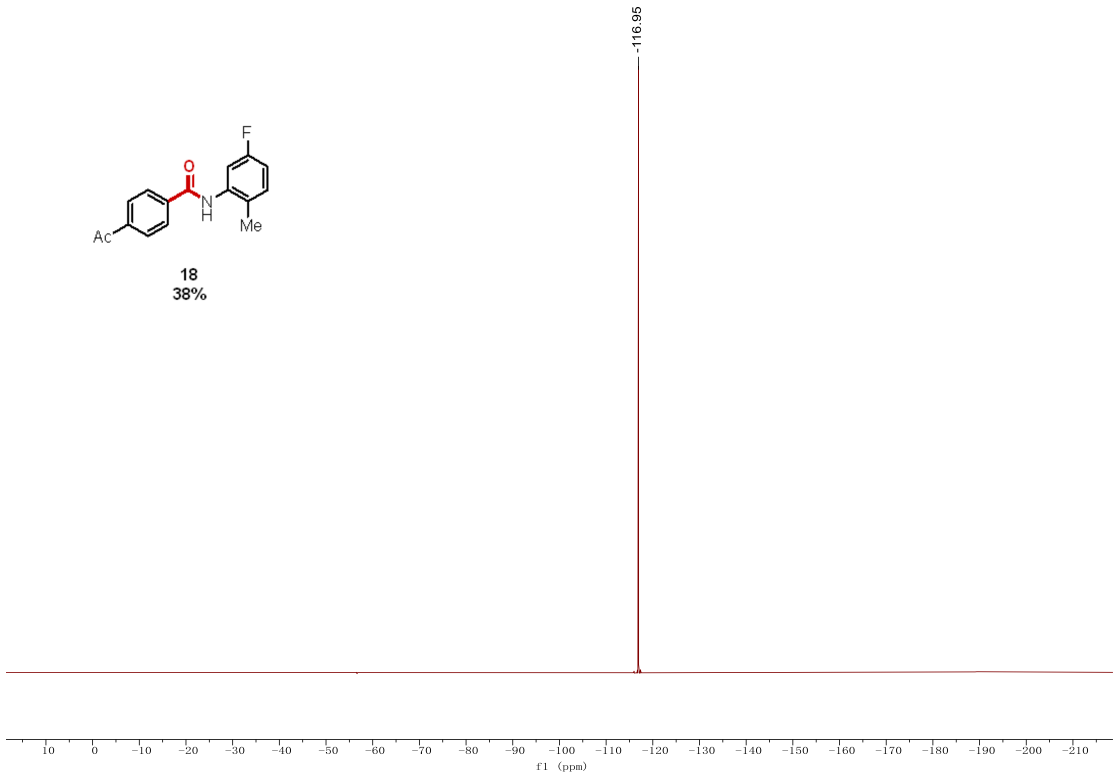

^{19}F NMR spectrum of **18** in DMSO-d_6 (376 MHz)

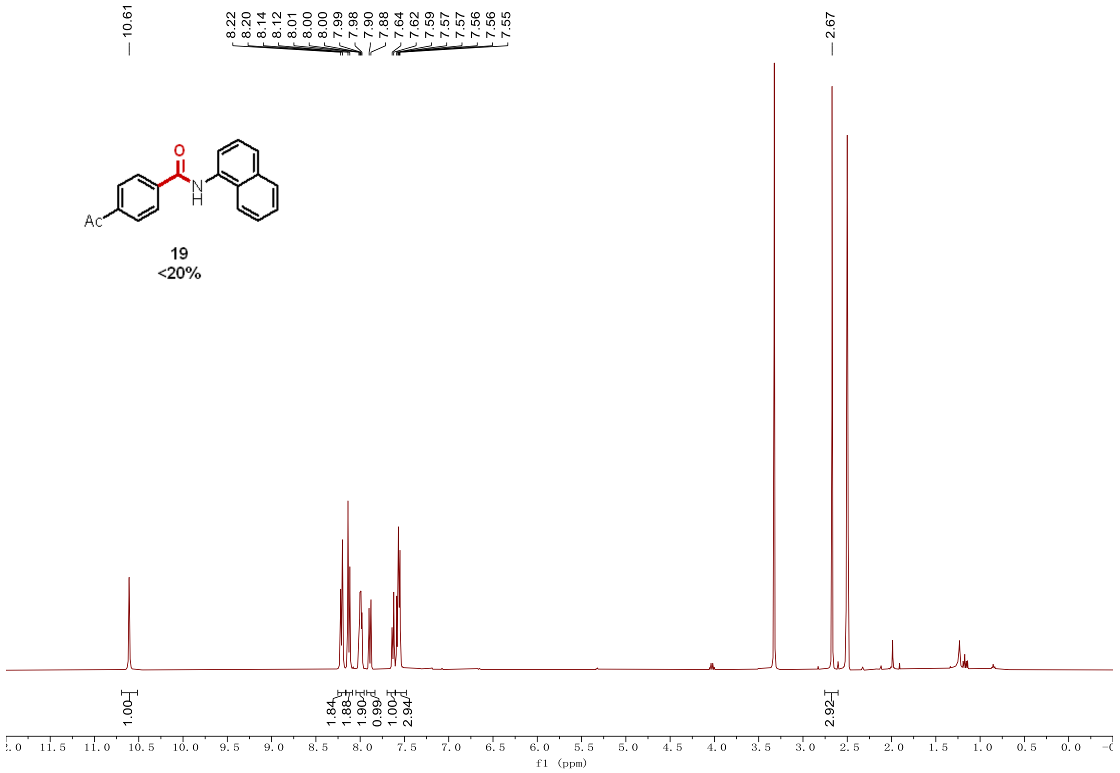

^{1}H NMR spectrum of **19** in DMSO-d_6 (400 MHz)

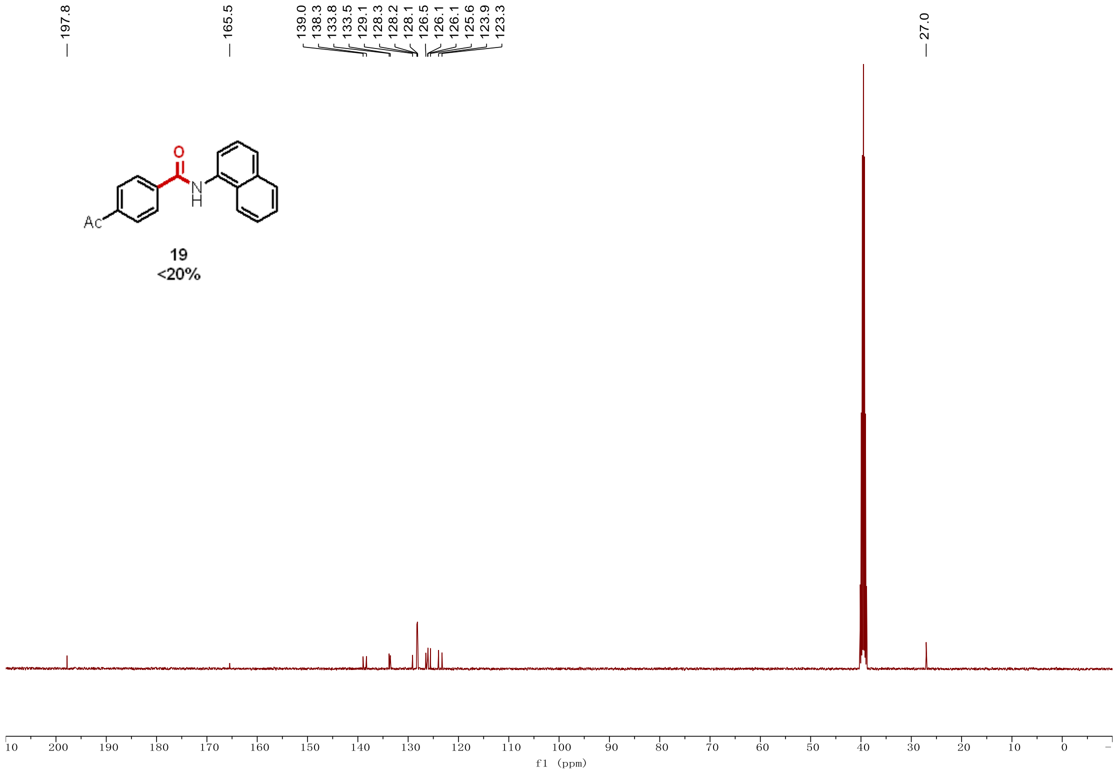

^{13}C NMR spectrum of **19** in DMSO-d_6 (101 MHz)

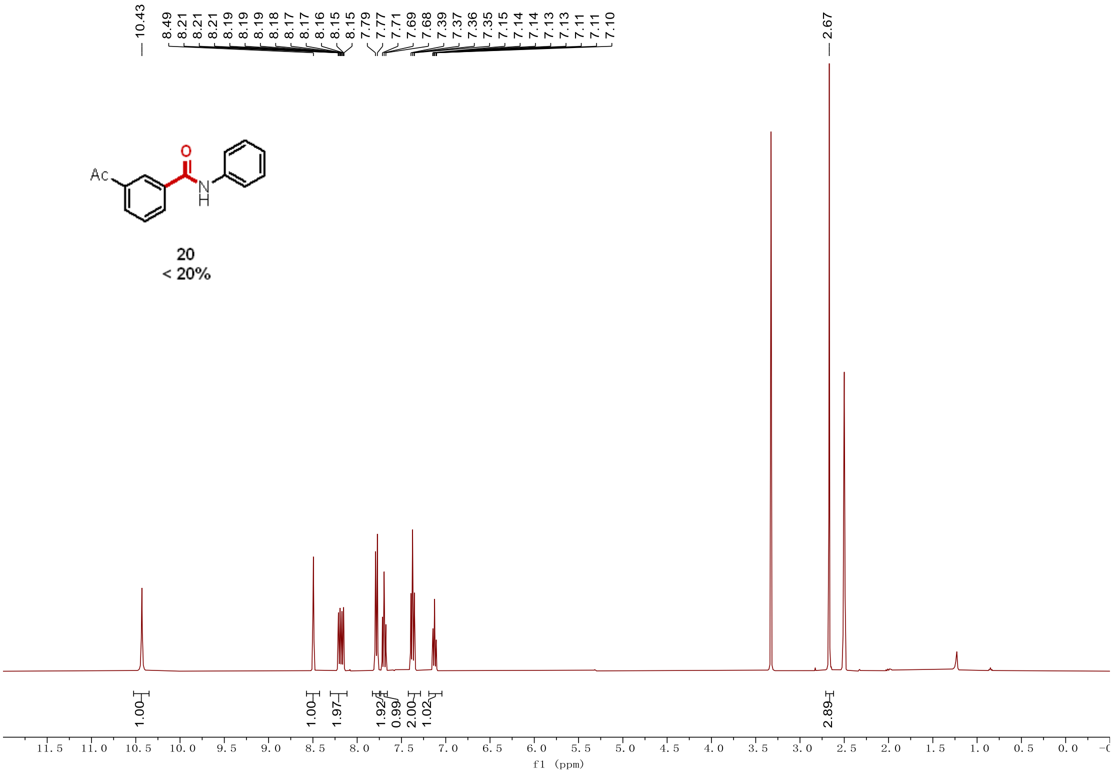

^{1}H NMR spectrum of **20** in DMSO-d_6 (400 MHz)

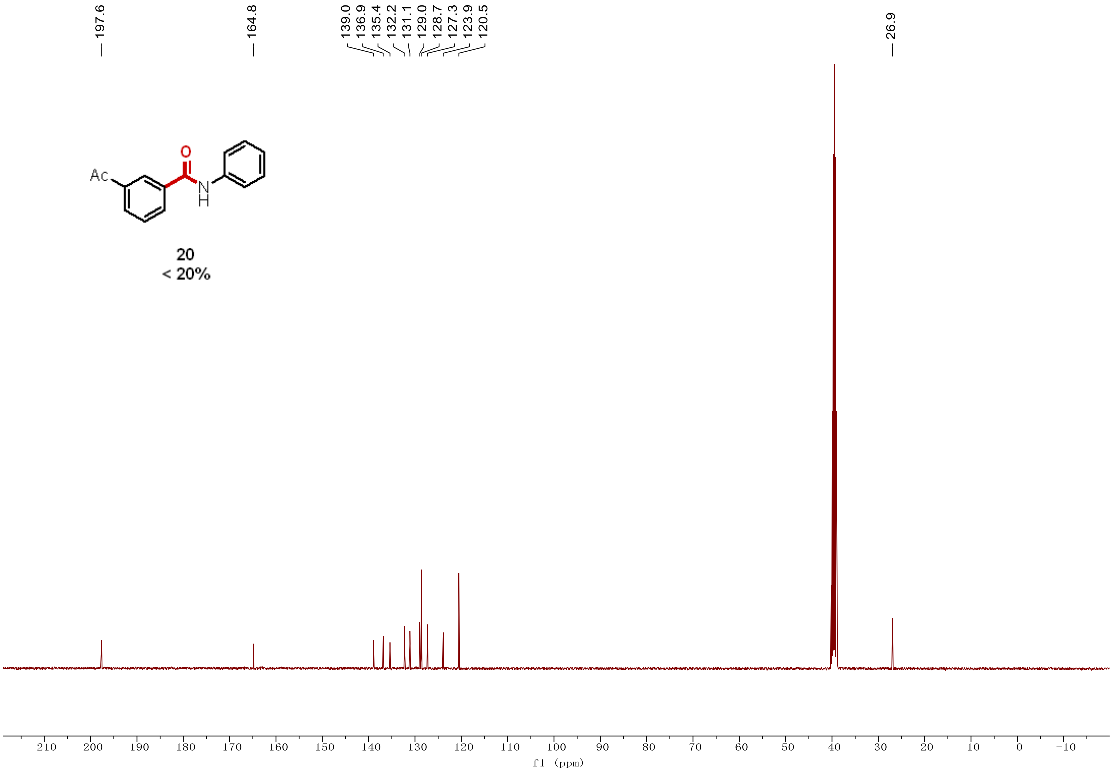

^{13}C NMR spectrum of **20** in DMSO-d_6 (101 MHz)
